# African Flora Has the Potential to Fight Multidrug Resistance of Cancer

**DOI:** 10.1155/2015/914813

**Published:** 2015-04-15

**Authors:** Victor Kuete, Thomas Efferth

**Affiliations:** ^1^Department of Pharmaceutical Biology, Institute of Pharmacy and Biochemistry, University of Mainz, Staudinger Weg 5, 55128 Mainz, Germany; ^2^Department of Biochemistry, Faculty of Science, University of Dschang, Dschang, Cameroon

## Abstract

*Background*. Continuous efforts from scientists of diverse fields are necessary not only to better understand the mechanism by which multidrug-resistant (MDR) cancer cells occur, but also to boost the discovery of new cytotoxic compounds to fight MDR phenotypes. *Objectives*. The present review reports on the contribution of African flora in the discovery of potential cytotoxic phytochemicals against MDR cancer cells. *Methodology*. Scientific databases such as PubMed, ScienceDirect, Scopus, Google Scholar, and Web of Knowledge were used to retrieve publications related to African plants, isolated compounds, and drug resistant cancer cells. The data were analyzed to highlight cytotoxicity and the modes of actions of extracts and compounds of the most prominent African plants. Also, thresholds and cutoff points for the cytotoxicity and modes of action of phytochemicals have been provided. *Results*. Most published data related to the antiproliferative potential of African medicinal plants were from Cameroon, Egypt, Nigeria, or Madagascar. The cytotoxicity of phenolic compounds isolated in African plants was generally much better documented than that of terpenoids and alkaloids. *Conclusion*. African flora represents an enormous resource for novel cytotoxic compounds. To unravel the full potential, efforts should be strengthened throughout the continent, to meet the challenge of a successful fight against MDR cancers.

## 1. Introduction

Cancer is increasingly recognized as a critical public health problem in Africa [1, 2]. The number of new cancer cases will reach 15 million every year by 2020 worldwide, 70% of which will be in developing countries, where governments are less prepared to address the growing cancer burden and where survival rates are often less than half of those in more developed countries [3]. Though communicable infections continue to burden African population, noncommunicable diseases also require the attention of health professionals in Africa [1]. Currently, limited funding is available to tackle cancer in African countries. Awareness of this impeding epidemic in Africa should be a priority today, and all possible resources should be mobilized to both prevent and efficiently treat cancers.

Cancer cells rapidly acquire multidrug resistance (MDR), mainly due to the presence of two adenosine triphosphate-binding cassette (ABC) transporters [4–6]. Continuous contributions of scientists from diverse fields are necessary not only to better understand the mechanisms of MDR, but also to boost the discovery of new cytotoxic drugs fighting drug resistance. The overexpression of ABC transporters contributes to MDR and participates in the failure of cancer chemotherapy [7]. MDR cancer cells reveal cross-resistance to a variety of chemically and functionally unrelated drugs [8–10]. The structural diversity of plant's secondary metabolites makes them an indispensable source for the discovery of new cytotoxic agents. Their use to combat drug resistance remains a challenging issue [11]. In the present review, we discuss the up-to-date prominent findings on anticancer plants and derived products from Africa.

## 2. Cancer Concern in Africa

Statistics of the International Agency for Research on Cancer (IARC) revealed that about 715,000 new cancer cases and 542,000 cancer deaths occurred in 2008 in Africa [12]. There will be about 1.28 million new cancer cases and 970,000 cancer deaths by 2030 solely in Africa, mainly due to aging and growth of the population [12]. The development might become even worse because of the adoption lifestyles associated with economic development, such as smoking, unhealthy diet, and physical inactivity [13]. The most occurring cancer types in Africa are those related to infectious agents (carcinoma of cervix, liver, and urinary bladder as well as Kaposi sarcoma) [1]. In 2008, cervical cancer accounted for 21% of the total newly diagnosed cancers in females and liver cancer for 11% of the total cancer cases in males [1]. The survival rates are considerably lower in Africa than in the developed world for most cancer types [1]. For example, the five-year survival rate for breast cancer is less than 50% in Gambia, Uganda, and Algeria, compared to nearly 90% in the United States [1]. According to the World Health Organization (WHO) government survey of national capacity for cancer control programs in 2001, anticancer drugs were only available in 22% and affordable in 11% of the 39 African countries that participated in the survey [2]. In parallel, efforts are being made by African scientists to search for new drugs from their most affordable resources, which are medicinal plants. Several plants from the flora of Africa were found to be active against various types of cancer cells. Even if not reported in the scientific literature for their antiproliferative potential, several medicinal plants of the African continent contain known antineoplastic compounds. Some of them include* Plumeria rubra* L. (Apocynaceae) with the well-reported cytotoxic compound plumericin or* Diospyros crassiflora* L. and* Diospyros canaliculata* L. (Ebenaceae) containing plumbagin [14]. Using a pharmacogenomics approach with Cameroonian flora as an example [14], it was demonstrated that African plants have an enormous and unstudied anticancer potential, as they contain an impressive arsenal of bioactive agents.

## 3. Cancer Cells and Drug Resistance

Cancer is caused by the accumulation of multiple genetic and epigenetic alterations, leading to abnormal expression of genes involved in initiation, progression, and promotion of carcinogenesis [15]. Cancer cells may rapidly acquire MDR, mainly due to the presence of adenosine triphosphate-binding cassette (ABC) transporters, such as the breast cancer resistance protein (BCRP/ABCG2) and P-glycoprotein (P-gp/MDR1/ABCB1) [4] as well as the oncogene epidermal growth factor receptor (EGFR) [5, 6, 16] and the deletions or inactivation of tumor suppressor gene p53 [17].

P-Glycoprotein 1 (permeability glycoprotein, P-gp or Pgp), encoded by the multidrug resistance gene 1 (*MDR1*), also known as ATP-binding cassette subfamily B member 1 (*ABCB1*) or cluster of differentiation 243 (CD243), is an important protein of the cell membrane that pumps many foreign substances out of cells [4]. It is an ATP-dependent efflux pump with broad substrate specificity found in animals, fungi, and bacteria and likely evolved as a defense mechanism against harmful substances during evolution of life. Some cancer cells overexpress P-gp rendering these cancers multidrug resistant [5, 6, 16, 18–20].

The human epidermal growth factor receptor (EGFR/ErbB-1/HER1) represents a cell-surface transmembrane glycoprotein that constitutes one of four members of the ErbB family of tyrosine kinase receptors [21]. EGFR is activated by binding of specific ligands, including epidermal growth factor and transforming growth factor *α* (TGF*α*). Upon activation, EGFR undergoes a transition from an inactive monomeric form to an active homodimer that stimulates its intrinsic intracellular protein-tyrosine kinase activity [22]. This leads to autophosphorylation of several tyrosine residues in the C-terminal domain of EGFR [23]. This autophosphorylation elicits downstream activation and signaling by several other proteins that associate with phosphorylated tyrosines through their own phosphotyrosine-binding SH_2_ domains, several signal transduction cascades (principally the MAPK, Akt, and JNK pathways) leading to DNA synthesis, and cell proliferation [24]. It was found that mutations leading to EGFR overexpression have been associated with many cancers, including lung cancer, anal cancers, and glioblastoma multiforme [25, 26].

Human tumor suppressor protein p53 (a protein of 53 kDa), also known as p53, cellular tumor antigen p53, and phosphoprotein p53, is encoded by the* TP53* gene [27]. The p53 protein is crucial in multicellular organisms, where it regulates the cell cycle and, therefore, functions as a tumor suppressor, preventing cancer [27]. Consequently p53 has been described as “the guardian of the genome” because of its role in conserving stability by preventing genome mutation, while* TP53* is classified as a tumor suppressor gene [27–29].

Topoisomerase (Topo) inhibitors are compounds interfering with the action of topoisomerase enzymes (Topo I and II), which are enzymes that control the changes in DNA structure by catalyzing the breaking and rejoining of the phosphodiester backbone of DNA strands during the normal cell cycle [30]. Topoisomerases have also become popular targets for cancer chemotherapy treatments, as their inhibitors block the ligation step of the cell cycle, generating single and double stranded breaks that harm the integrity of the genome leading to apoptosis and cell death [30].

## 4. Biodiversity and Protected Area in Africa

Considering biodiversity as genetic variation within populations, the number, relative abundance and uniqueness of species and the varieties, and finally the extent and condition of ecosystems are well endowed in Africa both in variety and in abundance of living organisms [31, 32]. Ecosystems are broadly arranged in latitudinal patterns with increasing species richness towards the equator. Plant species richness is also high in the winter-rainfall Mediterranean climate regions of Northern Africa and the Southern Cape. In between are the subtropical deserts, which are generally zones of lower diversity; for example, a vast part of the Sahara, the Ténéré, is home to only 20 plant species in an area of about 200,000 km^2^ [32]. In the latitudes from Ethiopia to the Cape, mountains contain several centers of endemism for birds, mammals, and plants. One of the globally most important centers of endemism is the coastal mountain range in the eastern part of Madagascar [32]. The increasing richness of plants and vertebrates toward the equator is related primarily to climatic factors, such as water availability. However the diversity of land variations, such as topography, is also important. There are exceptions to this: some areas with harsh climates, including the Namib Desert and the Karoo in the west of South Africa, have an estimated 4,500 plant species, a third to one-half of which are endemic. Overall plant richness at species, genus, and family level is lower than that of other tropical areas. The African mainland has between 40,000 and 60,000 plant species, of which approximately 35,000 are endemic [32]. South America, by comparison, has about 90,000 plant species in an area 40 percent smaller [32]. Parts of the Congo basin have moderate levels of plant species richness, comparable to many parts of Central Europe. Five of the 20 global centers of plant diversity are located in Africa. More than 3,000 plant species per 10,000 km^2^ occur in the Cameroon-Guinea center, the Capensis center, the Maputaland-Pondoland center, the Albertine Rift center, and the endemic Madagascar center [32]. At least a sixth of the world's plant species are in Africa. The Cape Floral Kingdom, a global center of plant endemism, has about 9,000 vascular plant species occurring in an area of 90,000 km^2^ of which about 69% are endemic. There are more than 12,000 plant species that occur in Madagascar [32]. At least 81% are endemic, which is an exceptionally high proportion by endemic global standards [32].

## 5. Anticancer Activity of Plants and Derived Products

Screenings of medicinal plants used as anticancer drugs have provided modern medicine with effective cytotoxic pharmaceuticals. More than 60% of the approved anticancer drugs in United States of America (from 1983 to 1994) were in one or another way from natural origin [33, 34]. The diversity of the biosynthetic pathways in plants has provided a variety of lead structures that have been used in drug development. In the past decade, investigations on natural compounds have been particularly successful in the field of anticancer drug research. Early examples of anticancer agents developed from higher plants are the antileukemic alkaloids (vinblastine and vincristine), which were both obtained from the Madagascar periwinkle (*Catharanthus roseus* L.; Apocynaceae) [35]. A large number of plant extracts have shown* in vitro* and* in vivo* antitumor activities [36].

For* in vitro* anticancer screenings of plant extracts, IC_50_ value of 30 *μ*g/mL represents a cutoff point to consider cytotoxic plant extracts for purification [36]. The IC_50_ value of 20 *μ*g/mL is also considered for good cytotoxic extract [36]. However, there is still a lack of scientific references to define other bioactivities, for example, unspecific toxicity towards normal cells as well as toxicity for edible or culinary plant's part. Herein, we will set values tenfold higher than 30 *μ*g/mL as the point of tolerably low cytotoxicity to normal cells and 10-fold higher than 20 *μ*g/mL as corresponding values in cancer cells. In this report, we propose the following cutoff points.

*(i) In Normal Cell Lines*. Significant or strong cytotoxicity: IC_50_ < 100 *μ*g/mL; moderate cytotoxicity: 100 *μ*g/mL < IC_50_ < 300 *μ*g/mL; low cytotoxicity: 300 *μ*g/mL < IC_50_ < 1000 *μ*g/mL; no cytotoxicity: IC_50_ > 1000 *μ*g/mL.

*(ii) In Cancer Cell Lines*. Significant or strong cytotoxicity: IC_50_ < 20 *μ*g/mL; moderate cytotoxicity: 20 *μ*g/mL < IC_50_ < 50 *μ*g/mL; low cytotoxicity: 50 *μ*g/mL < IC_50_ < 200 *μ*g/mL; no cytotoxicity: IC_50_ > 200 *μ*g/mL.

*(iii) If Dealing with Edible Parts of Plants, Culinary Plants, and Spices, We Define the following Thresholds Values in Cancer Cell Lines*. Significant or strong cytotoxicity: IC_50_ < 50 *μ*g/mL; moderate cytotoxicity: 50 *μ*g/mL < IC_50_ < 200 *μ*g/mL; low cytotoxicity: 200 *μ*g/mL < IC_50_ < 1000 *μ*g/mL; no cytotoxicity: IC_50_ > 1000 *μ*g/mL.

Similarly for plant metabolites, the following cutoff points are proposed.

*(iv) In Normal Cell Lines*. Significant or strong cytotoxicity: IC_50_ < 40 *μ*g/mL (or IC_50_ < 100 *μ*M); moderate cytotoxicity: 40 *μ*g/mL < IC_50_ < 120 *μ*g/mL (or 100 *μ*M < IC_50_ < 300 *μ*M); low cytotoxicity: 120 *μ*g/mL < IC_50_ < 400 *μ*g/mL (or 300 *μ*M < IC_50_ < 1000 *μ*M); no cytotoxicity: IC_50_ > 400 *μ*g/mL (or IC_50_ > 1000 *μ*M).

*(v) In Cancer Cell Lines*. Significant or strong cytotoxicity: IC_50_ < 4 *μ*g/mL (or IC_50_ < 10 *μ*M); moderate cytotoxicity: 4 *μ*g/mL < IC_50_ < 20 *μ*g/mL (or 10 *μ*M < IC_50_ < 50 *μ*M); low cytotoxicity: 20 *μ*g/mL < IC_50_ < 100 *μ*g/mL (or 50 *μ*M < IC_50_ < 250 *μ*M); no cytotoxicity: IC_50_ > 100 *μ*g/mL (or IC_50_ > 250 *μ*M).

## 6. Antiproliferative Effects of African Plants towards MDR Cancer Cells

Despite the exceptional biodiversity of Africa, few scientific studies have been carried out regarding the antiproliferative properties of medicinal plants. Nevertheless, efforts currently are being made, and some important results are continuously being reported on both medicinal plant extracts and compounds, especially in Cameroon, Egypt, Nigeria, and Madagascar. The majority of reports on MDR cancer cell lines were from Cameroonian plants and derived molecules. In this section, the state of the art of the most promising results will be provided.

### 6.1. Cytotoxicity of African Medicinal Spices

Several African medicinal spices were screened for their antiproliferative activities on both sensitive and resistant cancer cell lines (Table 1). The cytotoxicity of spices from* Aframomum *species (Zingiberaceae), namely,* Aframomum arundinaceum* (Oliv. & D. Hanb.),* Aframomum alboviolaceum (Ridl.)* K. Schum.,* Aframomum kayserianum* K. Schum, and* Aframomum polyanthum* K. Schum, was active at various degrees towards drug-resistant as well as sensitive cancer cells [37].* Aframomum polyanthum* and mostly* A. arundinaceum* demonstrated the best activities towards multidrug-resistant leukemia CEM/ADR5000 cells (degree of resistance (D.R.) or ratio of IC_50_ of resistant versus IC_50_ on corresponding sensitive cell line of 1.38 and 0.78, resp.), multidrug-resistant breast adenocarcinoma MDA-MB-231/*BCRP* cells (D.R.: 0.89 and 1.02, resp.), and glioblastoma multiforme U87MG.Δ*EGFR* cells (D.R.: <0.51 and 0.95, resp.) compared to their sensitive counterparts CCRF-CEM cells, MDA-MB-231 cells, and U87MG cells [37]. Interestingly, the extract from* A. arundinaceum* was less toxic to normal hepatocyte AML12 cells than to hepatocarcinoma HepG2 cells (D.R.: <0.58) [37]. Its active constituents were identified as galanals A (**2**) and B (**3**), naringenin (**7**), and kaempferol-3,7,4′-trimethylether (**8**) [37]. The antiproliferative effects of* Anonidium mannii* (Oliv.) Engl. (Annonaceae) harvested in Cameroon were also reported on various drug-resistant cancer cell lines (Table 1), with collateral sensitivity to the extract towards CEM/ADR5000 cells (D.R.: 0.95) and U87MG.Δ*EGFR* cells (D.R.: 0.41) [38]. Other Cameroonian spices, namely,* Xylopia aethiopica* (Dunal) A. Rich. (Annonaceae),* Echinops giganteus* A. Rich. (Asteraceae),* Imperata cylindrica* (L.) P. Beauv. (Poaceae), and* Piper capense* L.f. (Piperaceae), demonstrated a strong cytotoxicity towards a panel on sensitive and resistant cancer cell lines as shown in Table 1.* Xylopia aethiopica*,* Echinops giganteus,* and* Dorstenia psilurus* Welw. (Moraceae) showed strong effects on both leukemia CCRF-CEM and their drug-resistant subline CEM/ADR5000 cells (D.R.: 1.9, 1.2, and 1.1, resp.) [39]. Besides, other spices such as* Imperata cylindrica*,* Piper capense,* and* Zingiber officinale* Roscoe (Zingiberaceae) (Table 1) also displayed strong activities towards CCRF-CEM and CEM/ADR5000 cells with collateral sensitivity/hypersensitivity (degree of resistance below 1) [39]. BCRP-expressing MDA-MB-231 cells were reported to be 6.66-fold cross-resistant to the extract of* P. capense* but hypersensitive (collateral sensitive) to that of* Echinops giganteus*. Also, collateral sensitivity was observed with extracts from* Xylopia aethiopica*,* Echinops giganteus,* and* Piper capense* towards U87MG.Δ*EGFR* cells and with extracts from* E. giganteus* and* P. capense* against HCT116 (*p53* ^−/−^) cells [40]. Compounds such as 2-(penta-1,3-diynyl)-5-(4-hydroxybut-1-ynyl)-thiophene (**48**), candidone (**9**), and 4-hydroxy-2,6-di-(3′,4′-dimethoxyphenyl)-3,7dioxabicyclo-(3.3.0)octane (**10**) were identified as cytotoxic constituents of* Echinops giganteus* [40]. Other Cameroonian spices with hypersensitivity to CEM/ADR5000 compared to parental sensitive CCRF-CEM cells include* Olax subscorpioidea* Oliv. (Olacaceae),* Piper guineense* Schum. & Thonn. (Piperaceae),* Fagara leprieurii* (Guill. & Perr.) Engl. (Rutaceae), and* Aframomum melegueta* K. Schum. (Zingiberaceae) [39].

### 6.2. Cytotoxicity of Other African Plants on Drug-Resistant Cancer Cells

The fruit extract from the Cameroonian plant* Uapaca togoensis* Pax. (Euphorbiaceae) demonstrated a strong cytotoxicity on a panel of drug-resistant and -sensitive cancer cell lines [41]. Interestingly, collateral sensitivity to the extract was recorded towards P-gp-expressing CEM/ADR5000 cells, BCRP-expressing MDA-MB-231/*BCRP* cells, and U87MG.Δ*EGFR* cells [41]. The methanol extracts from* Gladiolus quartinianus* A. Rich. (Iridaceae) and* Vepris soyauxii* (Engl.) Mziray (Rutaceae), collected in Cameroon, were more cytotoxic to resistant U87MG.Δ*EGFR* cells compared to sensitive U87MG cells with degree of resistance below 0.85 and 0.47, respectively [38]. The bark extract of* Polyscias fulva* (Hiern) Harms. (Araliaceae) and leaves of* Beilschmiedia acuta* Kosterm (Lauraceae) also showed good activities towards drug-sensitive and -resistant cancer cell lines (Table 1), with lower degree of resistance than doxorubicin [20]. In fact, the hypersensitivity of colon carcinoma HCT116 (*p53* ^−/−^) cells to* Polyscias fulva* (D.R.: 0.41) and to* Beilschmiedia acuta *(D.R.: 0.23) extracts was reported [20]. In addition, these two extracts were more cytotoxic to the hepatocarcinoma HepG2 cells than to normal AML12 hepatocytes [20]. The medicinal plants* Crinum zeylanicum* Linn. (Amaryllidaceae),* Dioscorea bulbifera L.* (Dioscoreaceae),* Elaoephorbia drupifera* (Thonn.) Stapf. (Euphorbiaceae),* Entada abyssinica* Steud. ex A. Rich. (Mimosaceae),* Eremomastax speciosa* (Hochst) Cufod (Acanthaceae), and* Piliostigma thonningii* (Schum.) Milne-Redhead (Caesalpiniaceae) harvested in Cameroon were also investigated for their antiproliferative effects towards a panel of drug-sensitive and -resistant cancer cell lines (Table 1) [42]. The best activity was recorded with* Elaoephorbia drupifera*, which demonstrated a strong inhibitory effect on nine of nine tested cancer cell lines with other plants showing selective activities. Amongst these plants, the hypersensitivity of the resistant MDA-MB-231/*BCRP* cells (D.R. 0.62 compared to MDA-MB-231 cells) was observed towards* Crinum zeylanicum* extract and that of the resistant U87MG.Δ*EGFR* cells (D.R. 0.0.68 compared to U87MG cells) [42]. A panel of Egyptian medicinal plants was recently screened for their cytotoxic potential against four cell lines, namely, human pancreatic cancer MiaPaCa-2 cells, breast cancer MCF-7 cells, leukemia CCRF-CEM cells, and their multidrug-resistant subline CEM/ADR5000 cells [43]. They included* Ferula hermonis* Chirch el. (Apiaceae),* Bidens pilosa* L. (Asteraceae),* Crataegus sinaica* B. (Rosaceae),* Carduncellus eriocephalus* B. (Asteraceae),* Verbesina encelioides* (Cav.) Benth. & Hook. f. ex A. Gray (Asteraceae),* Carthamus tenuis* L. (Asteraceae),* Echinops spinosissimus* L. (Asteraceae),* Haplophyllum tuberculatum* (Forssk.) A. Juss. (Rutaceae),* Commiphora molmol* Jacq. (Burseraceae),* Cynara cornigera* (ssp.* sibthorbiana*) Lindl. (Asteraceae),* Cynara scolymus* L. (Asteraceae),* Camellia sinensis* (L.) Kuntze (Theaceae),* Cichorium intybus* L. (Asteraceae),* Foeniculum vulgare* var.* azoricum* (Apiaceae),* Foeniculum vulgare* (ssp.* piperitum*) (wild) (Apiaceae), and* Vitis vinifera* L. (Vitaceae). The study was then extended to the isolation of the antiproliferative compound from* F. hermonis.* Amongst these plants, only the extracts from* Crataegus sinaica*,* Carduncellus eriocephalus*,* Verbesina encelioides,* and* Carthamus tenuis* against CCRF-CEM and those from* Bidens pilosa*,* C. sinaica*,* C. tenuis*,* Haplophyllum tuberculatum,* and* Vitis vinifera* (brown seeds) against CEM/ADR5000 cells did not inhibit cancer cell growth [43]. All other extracts were able to inhibit the proliferation of CCRF-CEM as well as CEM/ADR5000 cells to various degrees. Only the fraction from* F. hermonis* and jaeschkeanadiol* p*-hydroxybenzoate (**4**), also known as ferutinin, isolated from its active fraction induced more than 50% inhibition against MiaPaCa-2 cells, MCF-7 cells, CCRF-CEM cells, and CEM/ADR5000 cells [43].

### 6.3. Cytotoxicity of Secondary Metabolites from African Plants towards MDR Cancer Cells

#### 6.3.1. Terpenoids

Terpenoids (or isoprenoids) are a large and diverse class of naturally occurring molecules derived from five-carbon isoprene units assembled and modified in thousands of ways. They are extraordinarily diverse in nature, but they all originate from condensation of universal phosphorylated derivatives of hemiterpene, isopentenyl diphosphate (IPP), and dimethylallyl diphosphate (DMAPP) giving geranyl pyrophosphate (GPP) [2]. They represent the most widespread group of natural products and can be found in all classes of living organisms. Terpenoids include monoterpenes (C10, e.g., carvone, geraniol, d-limonene, and perillyl alcohol) and sesquiterpenes (C15, e.g., farnesol) which are the main constituents of the essential oils as well as diterpenes (C20, e.g., retinol and trans-retinoic acid), sesterterpenes (C25), triterpenes (C30, e.g., betulinic acid, lupeol, oleanolic acid, and ursolic acid), and tetraterpenes (C40, e.g., *α*-carotene, *β*-carotene, lutein, and lycopene) [2]. Other terpenes are constituents of balsams, resins, waxes, and rubber [2]. Plant terpenoids play a role in traditional herbal remedies and were reported to have antibacterial, antimalarial, and antineoplastic activities and other pharmaceutical functions [2, 44]. However, some of them such as cicutoxin, atractyloside, daphnetoxin, digoxin, and gibberellic acid are involved in plant toxicity [45].

Many terpenoids with strong cytotoxic activity isolated in African medicinal plants were screened rather on drug-sensitive cancer cell lines. These include oleanane-type triterpenoid saponins; gummiferaosides A, B, and C (IC_50_ of 0.8, 1.5, and 0.6 *μ*g/mL, resp., on A2780 human ovarian cancer cells) obtained from the roots of the Madagascan plant* Albizia gummifera* J. F. Gmel. C. A. Sm. (Fabaceae) [46]; caseanigrescens A, B, C, and D (IC_50_ of 1.4, 0.83, 1.0, and 1.0 *μ*M, resp., against A2780 cancer cells) isolated from* Casearia nigrescens* (Flacourtiaceae) harvested in Madagascar [47]; cardenolide glycosides, elaeodendrosides V and W (IC_50_ of 0.12 and 0.07 *μ*M, resp., against A2780 cancer cells; 0.15 and 0.08 *μ*M against the U937 human histiocytic lymphoma cell line, resp.) isolated from another Madagascan plant* Elaeodendron alluaudianum* H. Perrier (Celastraceae) [48]; crotobarin (IC_50_ of 2.5; 2.1; 0.79; and 0.56 *μ*M, resp., against KB (human oral epidermoid carcinoma) cells, HT29 (human colon adenocarcinoma) cells, A549 (human lung adenocarcinoma) cells, and HL60 (human promyelocytic leukemia) cells); and crotogoudin (IC_50_ of 2.5; 2.1; 0.79; and 0.56 *μ*M, resp., against KB, HT29, A549, and HL60 cells) both isolated from* Croton barorum* Leandri and* Croton goudotii* Baill. (Euphorbiaceae) both of which were also collected in Madagascar [49].

**Figure 1:**
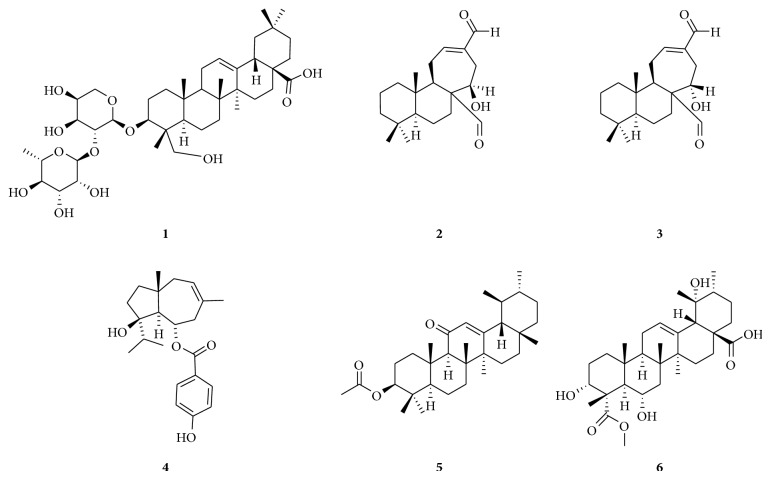
Cytotoxic terpenoids isolated from African medicinal plants with documented effects on MDR cancer cells. Alpha-hederin (**1**), galanal A (**2**), galanal B (**3**), jaeschkeanadiol* p*-hydroxybenzoate (**4**), 11-oxo-*α*-amyryl acetate (**5**), and elatunic acid (**6**).

Nonetheless, the inhibitory potential of few terpenoids towards drug-resistant cells was also reported (Figure 1). The bicyclic sesquiterpene esters jaeschkeanadiol* p*-hydroxybenzoate (**4**) isolated from the active fraction of the Egyptian medicinal plant* Ferula hermonis* exerted a strong cytotoxic effect towards breast cancer cell line MCF7 and moderate activities towards other cell lines (Table 1) [43]. However, this compound was as active on resistant leukemia CEM/ADR5000 cell line as towards its sensitive parental CCRF-CEM cell line, showing a degree of resistance of 1.06 [43]. The labdane diterpenoids galanals A (**2**) and B (**3**) (Figure 1) isolated from the Cameroonian spice* Aframomum arundinaceum* demonstrated moderate, but selective, cytotoxicity towards cancer cell lines [37] (Table 1). However, compounds** 2** and** 3** were generally less active towards resistant cancer cells, with** 3** showing collateral sensitivity towards resistant breast adenocarcinoma MDA-MB-231/*BCRP* cells (D.R.: <0.70 compared to its sensitive subline MDA-MB-231) [37].

A triterpenoid, 11-oxo-*α*-amyryl acetate (**5**) isolated from the fruits of* Uapaca togoensis* collected in Cameroon, displayed strong cytotoxicity towards sensitive leukemia CCRF-CEM cells, but cross-resistance was also noted towards CEM/ADR5000 cells [41]. A new ursolic acid-type from the Cameroonian plant* Omphalocarpum elatum* Miers. (Sapotaceae) named elatunic acid (**6**) showed moderate cytotoxic effects towards CCRF-CEM cells (IC_50_: 16.60 *μ*M) but low activity against CEM/ADR5000 cells (IC_50_: 67.91 *μ*M) [50]. The triterpene-saponin alpha-hederin (**1**) was identified as one of the cytotoxic constituents of* Polyscias fulva* with antiproliferative effects against both sensitive and resistant cell lines [20]. It showed collateral sensitivity towards HCT116 (*p53* ^−/−^) and HepG2 as compared to the sensitive counterparts HCT116 (*p53* ^+/+^) and the normal AML12 hepatocytes [20]. In similar experimental conditions with doxorubicin, compound** 1** showed lower cross-resistance towards CEM/ADR5000 cells (D.R.: 2.91 for** 1** and 975.60 for doxorubicin), MDA-MB-231/*BCRP* cells (D.R.: 5.09 for** 1** and 7.11 for doxorubicin), and glioblastoma multiforme U87MG.Δ*EGFR* cells (D.R.: 3.94 for** 1** and 5.76 for doxorubicin) as compared to their sensitive sublines CCRF-CEM, MDA-MB231, and U87MG, respectively [20]. These data suggested that though compound** 1** was generally less active than doxorubicin, it can be helpful in the management of resistant forms of cancers.

#### 6.3.2. Phenolics

The term phenolic compound or phenolic is applied to several classes of aromatic compounds such as simple phenol, phenolic acids, phenylpropanoids, flavonoids, coumarins, quinones, xanthones, anthrones, lignans, stilbenes, and tannins [2]. Phenolic compounds exert a variety of biological activities, including anti-inflammatory, anticancer, antimicrobial, antimalarial, and antioxidant effects [2]. However, some of them such as chamuvaritin, gossypol, plumbagin, and scopoletin were also documented as toxic principles of some medicinal plants [51].

**Figure 2:**
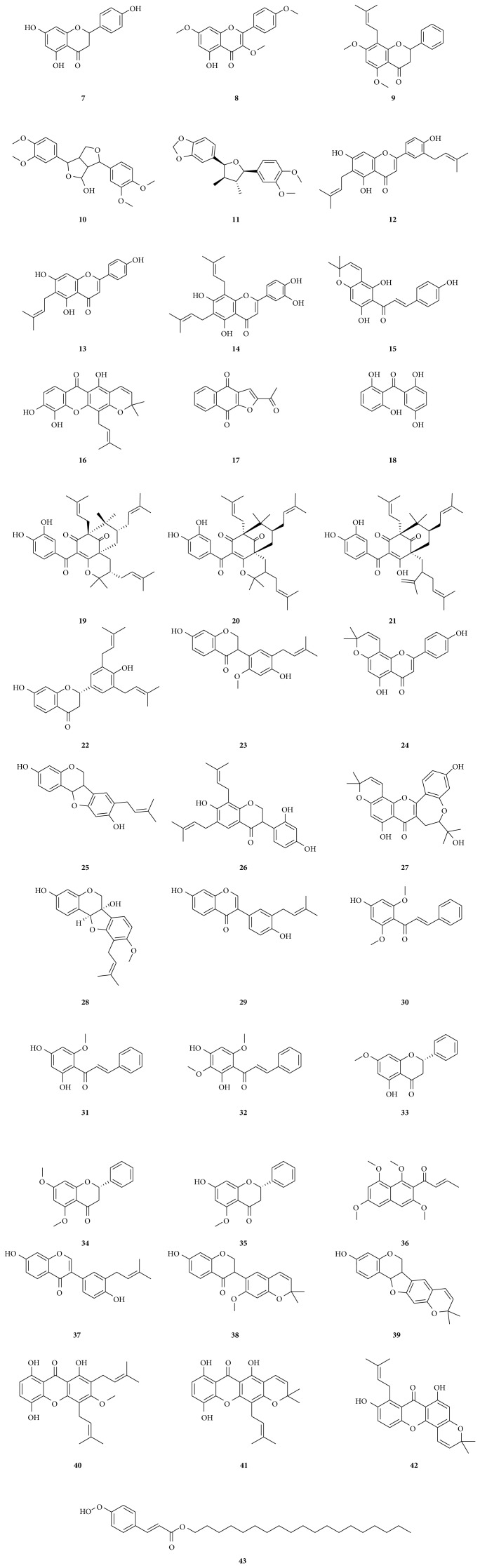
Cytotoxic phenolics isolated from African medicinal plants with documented activity on MDR cancer cells. Naringenin (**7**), kaempferol-3,7,4′-trimethylether (**8**), candidone (**9**), 4-hydroxy-2,6-di-(3′,4′-dimethoxyphenyl)-3,7dioxabicyclo-(3.3.0)octane (**10**), futokadsurin B (**11**), gancaonin Q (**12**), 6-prenylapigenin (**13**), 6,8-diprenyleriodictyol (**14**), 4-hydroxylonchocarpin (**15**), xanthone V_1_ (**16**), 2-acetylfuro-1,4-naphthoquinone (**17**), 2,2′,5,6′-tetrahydroxybenzophenone (**18**), isogarcinol (**19**), isoxanthochymol (**20**), guttiferone E (**21**), abyssinone IV (**22**), sigmoidin I (**23**), atalantoflavone (**24**), sophorapterocarpan A (**25**), bidwillon A (**26**), neocyclomorusin (**27**), 6*α*-hydroxyphaseollidin (**28**), neobavaisoflavone (**29**), 4′-hydroxy-2′,6′-dimethoxychalcone (**30**), cardamomin (**31**), 2′,4′-dihydroxy-3′,6′-dimethoxychalcone (**32**), (*S*)-(–)-pinostrobin (**33**), (*S*)-(–)-onysilin (**34**), alpinetin (**35**), guieranone A (**36**), neobavaisoflavone (**37**), sigmoidin H (**38**), isoneorautenol (**39**), 8-hydroxycudraxanthone G (**40**), morusignin I (**41**), cudraxanthone I (**42**), and excelsaperoxide (**43**).

A number of phenolic compounds from African plant were reported for their effects towards sensitive as well as drug-resistant cancer cell lines (Figure 2). Some of the most active ones include the flavanone naringenin (**7**) and the flavonol kaempferol-3,7,4′-trimethylether (**8**) isolated from* Aframomum arundinaceum* collected in Cameroon [37] (Table 1) and compound** 7** having the best spectrum of activity. Though the activities of the two compounds were moderate towards the majority of the reported cell lines, they displayed rather lower cross-resistance (D.R.: 0.60 to 1.91) compared to doxorubicin (D.R.: 2.84 to 1772) [37] on CEM/ADR5000 cells, MDA-MB-231/BCRP cells, HCT116 (*p53* ^−/−^) cells, and U87MG.Δ*EGFR* cells [37]. Another flavanone named candidone (**9**) and a lignan known as 4-hydroxy-2,6-di-(3′,4′-dimethoxyphenyl)-3,7dioxabicyclo-(3.3.0)octane (**10**) isolated from the active fractions on the Cameroonian spice* Echinops giganteus* were active towards resistant HL60AR cells and HCT116 (*p53* ^−/−^) cells. However, the documented activities were low with IC_50_ values varying from 32 to 39 *μ*g/mL [40]. Also, futokadsurin B (**11**), a lignan isolated from* Uapaca togoensis,* displayed a strong activity, combined with hypersensitivity against leukemia CEM/ADR5000 cells compared to CCRF-CEM cells [41].

Flavonoids from the genus* Dorstenia*, gancaonin Q (**12**), 6-prenylapigenin (**13**), 6,8-diprenyleriodictyol (**14**), and 4-hydroxylonchocarpin (**15**) inhibited the proliferation of a panel of 14 cancer cell lines, including human leukemia CCRF-CEM cells and CEM/ADR5000, leukemia T cells PF-382, and promyelocytic leukemia HL-60, pancreatic adenocarcinoma MiaPaCa-2 and Capan-1, breast adenocarcinoma MCF-7, colon carcinoma SW-680, renal carcinoma 786-0, glioblastoma-astrocytoma U87MG, lung adenocarcinoma A549, cervical carcinoma CaSki and HeLa, and skin melanoma Colo-38 cells [52]. IC_50_ values below or around 4 *μ*g/mL were reported for compound** 12** on PF-382 and HL-60 cells (4.8 *μ*g/mL), MiaPaCa-2 cells (1.1 *μ*g/mL), and MCF-7 cells (0.8 *μ*g/mL), compound** 13** on PF-382 cells (3.8 *μ*g/mL) and MCF-7 cells (0.6 *μ*g/mL), compound** 14** on CCRF-CEM cells (4.9 *μ*g/mL), MiaPaCa-2 cells (4.4 *μ*g/mL), and MCF-7 cells (0.6 *μ*g/mL), and compound** 15** on CCRF-CEM cells (1.6 *μ*g/mL), CEM/ADR5000 cells (3.7 *μ*g/mL), MiaPaCa-2 cells (3.8 *μ*g/mL), and MCF-7 cells (1.4 *μ*g/mL) [52]. Hypersensitivity of CEM/ADR5000 cells to compound** 12** (D.R.: 0.66 compared with CCRF-CEM cells) and cross-resistance to compounds** 13**,** 14**, and** 15** were reported [52]. Several phenolic compounds isolated from the bark of* Erythrina sigmoidea* Hua (Fabaceae) collected in Cameroon, namely, the flavonoids abyssinone IV (**22**), atalantoflavone (**24**), neocyclomorusin (**27**), the isoflavonoids sigmoidin I (**23**), and bidwillon A (**26**) as well as the pterocarpan isoflavonoids sophorapterocarpan A (**25**), 6*α*-hydroxyphaseollidin (**28**), and neobavaisoflavone (**29**) also displayed strong to moderate activities against drug-sensitive and -resistant leukemia, breast, colon, and glioblastoma carcinoma cells lines [53]. Interestingly, P-gp-expressing CEM/ADR5000 as well as p53-knockout HCT116 (*p53* ^−/−^) and U87MG.Δ*EGFR* cells were less cross-resistant towards the phytochemicals** 22**–**29** than towards doxorubicin [53]. The cytotoxicity of three isoflavonoids also* Erythrina excelsa* Bak. and* Erythrina senegalensis* DC., compound** 29**, sigmoidin H (**38**), and isoneorautenol (**39**) towards a panel multifactorial drug-resistant cancer cells was documented too [54]. However, the activities of compounds** 29** and** 38** were rather moderate; meanwhile strong activity was recorded with** 39** towards CCRF-CEM cells (IC_50_: 7.51 *μ*M), MDA-MB-231/*BCRP* cells (IC_50_: 2.67 *μ*M), and HCT116 (*p53* ^+/+^) cells (IC_50_: 9.89 *μ*M) [54]. In addition, BCRP-transfected MDA-MB-231 cells, HCT116 (*p53* ^+/+^), and U87MG.Δ*EGFR* cells were found to be hypersensitive to** 39** as compared to their parental cell lines [54].

The antiproliferative activities of three xanthones, namely, 8-hydroxycudraxanthone G (**40**) and morusignin I (**41**) isolated from* Garcinia nobilis* Engl. (Guttiferae) and cudraxanthone I (**42**) isolated from* Milicia excelsa* Welw C.C. Berg. (Moraceae) collected in Cameroon, were also documented on several drug-sensitive and -resistant cancer cells [55]. The reported effects were observed in more than half of the nine studied cell lines with IC_50_ values ranging from 16.65 *μ*M to 70.38 *μ*M (against HepG2 cells) for** 40**, from 7.15 *μ*M to 53.85 *μ*M for** 41**, and from 2.78 *μ*M to 22.49 *μ*M for** 42** [55]. BCRP-expressing MDA-MB-231 cells were hypersensitive to** 40** and** 42** [55]. However, HCT116 (*p53* ^−/−^) cells were 1.33- and 1.36-fold resistant to** 41** and** 42**, respectively, but collaterally sensitive to** 40** compared to HCT116 (*p53* ^+/+^) [55]. Furthermore, collateral sensitivities were observed for** 40** and** 42** in U87MG.Δ*EGFR* cells compared to wild-type U87MG cells, but cross-resistance (2.01-fold) was noted for** 41** [55].

Xanthone V_1_ (**16**) isolated from the Cameroonian plant* Vismia laurentii* De Wild (Guttiferae) and 2-acetylfuro-1,4-naphthoquinone (**17**) isolated from* Newbouldia laevis* Seems (Bignoniaceae) were also tested on the above panel of 14 cell lines [18]. IC_50_ values below or around 4 *μ*g/mL were reported for** 16** on CCRF-CEM cells (4.9 *μ*g/mL), HL60 cells (4.56 *μ*g/mL), 786-0 cells (3.79 *μ*g/mL), U87MG cells (3.80 *μ*g/mL), A549 cells (3.99 *μ*g/mL), Colo-38 cells (1.19 *μ*g/mL), and CaSki cells (0.24 *μ*g/mL) [18]. IC_50_ values below or around 4 *μ*g/mL were also reported with compound** 17** on PF-382 cells (0.57 *μ*g/mL), Colo-38 cells (0.67 *μ*g/mL), HeLa cells (0.40 *μ*g/mL), and CaSki cells (0.17 *μ*g/mL) [18]. Cross-resistance of CEM/ADR5000 cells as compared to CCRF-CEM cells was also noted towards** 16** and** 17** [18].

Four naturally occurring benzophenones 2,2′,5,6′-tetrahydroxybenzophenone (**18**), isogarcinol (**19**) isolated from* Hypericum lanceolatum* Lam. (Hypericaceae), isoxanthochymol (**20**), and guttiferone E (**21**) isolated from the* Garcinia punctata* Oliv. (Guttiferae) collected in Cameroon demonstrated strong antiproliferative effects towards a panel of sensitive and drug-resistant hematological, breast, colon, and glioblastoma cancer cell lines [19]. The hypersensitivity of glioblastoma U87MG.Δ*EGFR* cells compared to its parental drug-sensitive cell line U87MG was recorded towards four benzophenones [19]. Also, the breast adenocarcinoma MDA-MB-231/*BCRP* cells were hypersensitive to** 19** and** 20** (compared to MDA-MB-231 cells). Meanwhile collateral sensitivity was also observed with the p53-knockout colon HCT116 (*p53* ^−/−^) cells towards** 21** (compared to HCT116 cells) [19]. The ability of other flavonoids from the Cameroonian plant,* Polygonum limbatum* Meisn. (Polygonaceae), to tackle MDR of cancer cells was also reported. These flavonoids included three chalcones [4′-hydroxy-2′,6′-dimethoxychalcone (**30**), cardamomin (**31**), and 2′,4′-dihydroxy-3′,6′-dimethoxychalcone (**32**)] and three flavanones [(*S*)-(–)-pinostrobin (**33**), (*S*)-(–)-onysilin (**34**), and alpinetin (**35**)] [56]. The best activity was noted with compound** 30**, with IC_50_ values <10 *μ*M in more than 50% of the studied cell lines, including the resistant BCRP-transfectant MDA-MB-231 cells (6.48 *μ*M) and the p53-knockout HCT116 cells (6.27 *μ*M) [56]. Besides, collateral sensitivity was observed in CEM/ADR5000 cells, MDA-MB-231/*BCRP* cells, p53-knockout HCT116 cells, and HepG2 cells and only weak cross-resistance in U87MG.Δ*EGFR* cells [56]. In addition, the CEM/ADR5000 cells showed much more sensitivity towards** 30** than towards doxorubicin [56].

The naphthyl butenone guieranone A (**36**) isolated from* Guiera senegalensis* J. F. Gmel. (Combretaceae) was active on 11 of 12 cancer cell lines. IC_50_ values below 10 *μ*M were recorded in CCRF-CEM cells (2.31 *μ*M) and on CEM/ADR5000 cells (3.19 *μ*M), MCF-7 cells (3.42 *μ*M), U87MG cells (7.78 *μ*M), A549 cells (2.28 *μ*M), HeLa cells (1.61 *μ*M), and CaSki cells (3.73 *μ*M) [57]. In addition to low cross-resistance of CEM/ADR5000 cells, compound** 36** was less toxic to the normal hepatocytes AML12 cells, highlighting its selectivity [57].

A new cinnamate derivative obtained from* Erythrina excelsa* Bak. (Fabaceae) and identified as* para*-hydroperoxycoumaroate of nonadecyl or excelsaperoxide (**43**) displayed significant activity against resistant leukemia CEM/ADR5000 cells (IC_50_: 1.07 *μ*M) and CCRF-CEM cells (IC_50_: 1.02 *μ*M) [58]. In addition, this compound also showed strong activities towards MDA-MB-231 cells (IC_50_: 3.22 *μ*M) and U87MG cells (IC_50_: 3.75 *μ*M) but low effects towards HCT116 (*p53* ^+/+^) cells and HepG2 cells (IC_50_: 57.77 *μ*M) [58].

#### 6.3.3. Alkaloids

Alkaloids are one of the most diverse groups of secondary metabolites found in plants, marine organisms, and microorganisms [2]. A well accepted definition is that alkaloids are naturally occurring, nitrogen-containing organic compounds with the exception of amino acids, peptides, purines and derivatives, amino sugars, and antibiotics. The nitrogen atom remains as a heterocyclic ring with some exceptions. Based upon biogenesis, the alkaloids are broadly classified as* true alkaloids* with heterocyclic nitrogen atom and* pseudo alkaloids*. They have an array of structural type, biosynthetic pathways, and pharmacological activities [2]. It is also worth noting that some alkaloids including anabasine, aristolochic acid I, nicotine, sanguinarine, and solanine are involved in plant side effects to humans and animals [59, 60].

Compared to terpenoids and phenolics, a limited number of alkaloids isolated from African medicinal plants were reported for their cytotoxic effects on cancer cells. However, data available from the screening of some compounds isolated from African plants are rather moderate even when sensitive cell lines are involved. This is the case with the acridone alkaloids isolated from the fruits of* Zanthoxylum leprieurii* Guill. & Perr. (Rutaceae) collected in Cameroon, namely, helebelicine A, 3-hydroxy-1-methoxy-10-methyl-9-acridone, 1-hydroxy-3-methoxy-10-methyl-9-acridone, and 1-hydroxy-2,3-dimethoxy-10-methyl-9-acridone that showed moderate activity against human lung carcinoma cells A549 (IC_50_ values of 31 to 52 *μ*M) and colorectal adenocarcinoma cells DLD-1 (IC_50_ of 27 to 74 *μ*M) [61].

**Figure 3:**
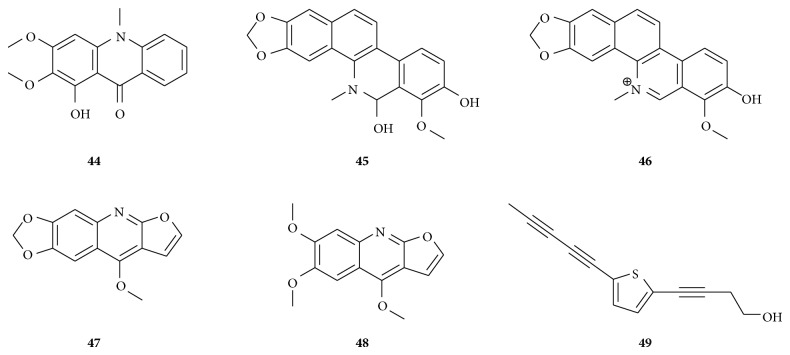
Cytotoxic Alkaloids (**44**–**49**) and a thiophene (**49**) isolated from African medicinal plants with relevance to MDR cancer cells. Arborinin (**44**), buesgenine (**45**), isofagaridine (**46**), maculine (**47**), kokusaginine (**48**), and 2-(penta-1,3-diynyl)-5-(4-hydroxybut-1-ynyl)-thiophene (**49**).

However, another acridone alkaloid arborinin (**44**) (Figure 3) from* Uapaca togoensis* displayed strong activities in several cancer cell lines, including MDR phenotypes (Table 1); importantly, hypersensitivity to** 44** was reported with CEM/ADR5000 cells (D.R.: 0.11 compared to CCRF-CEM cells), MDA-MB-231/*BCRP* cells (D.R.: 0.87 compared to MDA-MB-231 cells), and U87MG.Δ*EGFR* (D.R.: 0.34 compared to U87MG cells) [41].

Four alkaloids including two benzophenanthridines, buesgenine (**45**) and isofagaridine (**46**), and two fluoroquinolones, maculine (**47**) and kokusaginine (**48**), isolated from the aerial part of the Cameroonian spice* Zanthoxylum buesgenii* Engl. (Rutaceae) showed antiproliferative effects on a panel of drug-sensitive and -resistant cancer cell lines [62]. Nevertheless, the reported activities were rather moderate or low, even though** 45** and** 46** had broad cytotoxicity spectra [62]. However, strong activities towards CCRF-CEM cells were recorded with** 45** (IC_50_: 24 *μ*M) and** 46** (IC_50_: 0.30 *μ*M) [62].

#### 6.3.4. Cytotoxicity of the Thiophene 2-(Penta-1,3-diynyl)-5-(4-hydroxybut-1-ynyl)-thiophene

Though the thiophene isolated from the roots of* Echinops giganteus*, 2-(penta-1,3-diynyl)-5-(4-hydroxybut-1-ynyl)-thiophene (**49**), demonstrated a broad spectrum of cytotoxic activities including resistant cancer cells [40], its inhibitory potential was found to be moderate (IC_50_ range: 19–38 *μ*g/mL). However, hypersensitivity of HCT116 (*p53* ^−/−^) (compared to HCT116 (*p53* ^+/+^)) to this compound was reported [40].

## 7. Mode of Action of African Plant Extracts and Derived Products with Cytotoxic Effect on Drug-Resistant Cancer Cell Lines

The mode of action of many African plant extracts and isolated compounds having good antiproliferative activities on drug-resistant cells has been demonstrated. The documented modes of induction of apoptosis include activation of caspases, alteration of mitochondrial membrane potential (MMP), generation of reactive oxygen species (ROS), and inhibition of angiogenesis. In this section, the synopsis of these mechanistic data will be provided.

### 7.1. Induction of Apoptosis and Cell Cycle Arrest

Several African plant extracts and isolated compounds acting on MDR cancer phenotypes were found to induce apoptosis and cell cycle arrest in cancer cells. In this review, we proposed to classify the induction of apoptosis by plant extracts or derived molecules at not more than their twofold IC_50_ values as follows: (i) very strong: if the percentage of induction is above 50%; (ii) strong: if the percentage of induction is between 20 and 50%; (iii) moderate: if the percentage of induction is between 10 and 20%; (iv) low: if the percentage of induction is between 4 and 10%; and (v) no induction: if the percentage of induction is below 4%.

The reported African medicinal plants with significant cytotoxic effects on MDR cancer cells and showing very strong induction of apoptosis include* Echinops giganteus, Imperata cylindrica, Piper capense* [40],* Gladiolus quartinianus, Vepris soyauxii,* and* Anonidium mannii* [38]. A moderate to strong induction of apoptosis was also recorded with the spice of* Xylopia aethiopica* [40]. It was also shown that most of the crude extracts from African medicinal plants induced cell cycle arrest mostly in G0/G1 and between G0/G1 and S phases. In fact, the cell cycle arrest in G0/G1 in leukemia CCRF-CEM cells was reported with the extracts from* Vepris soyauxii*,* Anonidium mannii* [38],* Echinops giganteus,* and* Piper capense* [40]. Arrest between G0/G1 and S phases in CCRF-CEM cells was reported with the extracts from* Gladiolus quartinianus* [38],* Imperata cylindrica, Xylopia aethiopica* [40],* Polyscias fulva,* and* Beilschmiedia acuta* [20].

Compounds such as benzophenones** 19**,** 20**, and** 21** demonstrated a very strong induction of apoptosis in leukemia CCRF-CEM cells [19]; benzophenones** 19** and** 21** did not induce cell cycle arrest in either G0/G1, S, or M phases, but compound** 20** induced arrest in G0/G1 phase [19]. Flavonoids** 22** and** 30** as well as pterocarpan isoflavonoids** 25**,** 28**, and** 39** and the naphthyl butenone** 36** also induced cell cycle arrest in G0/G1 phase, whilst the isoflavonoid** 23**, the acridone alkaloid** 44**, induced arrest between G0/G1 and S phases [41, 53, 54, 56, 57]. A strong induction of apoptosis was also recorded with** 22**,** 23**,** 25**, and** 28** and the xanthone** 42** on CCRF-CEM cells [53, 55]. However, cell cycle arrest in S phase was also reported with xanthone** 16** and naphthoquinone** 17** in CCRF-CEM cells [18].

### 7.2. Effects of African Plant Extract and Derived Molecules on Caspase Activation

Caspases, a family of cysteine proteases, are central regulators of apoptosis [63]. Initiator caspases (caspases 2, 8, 9, 10, 11, and 12) are closely coupled to proapoptotic signals [63]. Upon activation, initiator caspases cleave and activate downstream effector caspases (caspases 3, 6, and 7), which in turn execute apoptosis by cleaving cellular proteins at specific aspartate residues [63]. In this report, we propose to classify the activation of caspases by plant extracts or derived molecules at not more than their twofold IC_50_ values which will be as follows: (i) very high: if the increase is more than 64-fold; (ii) high: if the increase is between 8- and 64-fold; (iii) moderate: if the increase is between 4- and 8-fold; (iv) low: if the induction is 1–4-fold; and (v) no induction: if the induction is less than 1-fold.

In general, several crude extracts having inhibitory effect on MDR cancer cells were reported not to induce the activation of caspase enzymes [20, 40]. Benzophenones** 19**–**21** were able to activate caspases in CCRF-CEM cells treated with concentrations equivalent to their IC_50_ values [19]. A high activation was observed for caspases 3/7, whereas the effects on caspases 8 and 9 were moderate [19]. A high activation of caspases 3/7 activity and moderate activation of caspases 8 and 9 were also reported with the pterocarpan** 39** as well as the xanthones** 16** and** 42** [18, 54, 55]. Low activation of caspases 3/7, 8, and 9 was reported with the pterocarpan** 28**, whilst no effect was obtained in similar experimental condition with** 22**,** 23**, and** 25** [54].

### 7.3. Effects of African Plant Extract and Derived Molecules on the Mitochondrial Membrane Potential

Apoptotic proteins target mitochondria and affect them in different ways. If cytochrome c is released from mitochondria due to formation of a channel in the outer mitochondrial membrane during the apoptosis process, it binds to apoptotic protease activating factor-1 (Apaf-1) and ATP, which then bind to procaspase-9 creating a protein complex known as apoptosome [64]. Herein, we propose to classify the extent of MMP alteration by plant extracts or derived molecules at not more than their twofold IC_50_ values as follows: (i) very strong: if the percentage of MMP disruption is more than 50%; (ii) strong: if the percentage of MMP disruption is between 20 and 50%; (iii) moderate: if the percentage of MMP disruption is between 10 and 20%; (iv) low: if the percentage of MMP disruption is between 5 and 10%; and (v) no induction: if the percentage of induction is below 5%.

MMP disruption in cancer cells was reported as one of the likely mechanisms of induction of apoptosis by several African plant extracts and derived compounds. A strong depletion of MMP in CCRF-CEM cells was reported with crude extracts from* Echinops giganteus, Xylopia aethiopica *[40], and* Anonidium mannii* [38]. Moderate alterations of the MMP in CCRF-CEM cells were measured with extracts from* Imperata cylindrica* and* Piper capense* [40],* Gladiolus quartinianus*,* Vepris soyauxii* [38], and* Polyscias fulva* [20].

Benzophenones** 19**–**21**, flavonoid** 22**; isoflavonoids** 19** and** 23**; and compounds** 28** and** 30** as well as xanthone** 42** strongly disrupted MMP in CCRF-CEM cells in a dose-dependent manner [19, 53, 55, 56].

### 7.4. Effects of African Plant Extract and Derived Molecules on Generation of Reactive Oxygen Species

The appearance of malignancies resulting in gain-of-function mutations in oncogenes and loss-of-function mutations in tumour suppressor genes leads to cell deregulation that is frequently associated with enhanced cellular stress [65, 66]. In the present paper, we recommend to classify the extent of ROS production by plant extracts or derived molecules at not more than their twofold IC_50_ values as follows: (i) very high: if the percentage of ROS production is more than 50%; (ii) high: if the percentage of ROS production is between 20 and 50%; (iii) moderate: if the percentage of ROS production is between 10 and 20%; (iv) low: if the percentage of ROS production is between 3 and 10%; and (v) no induction: if the percentage of ROS production is below 3%.

Increased ROS production in leukemia CCRF-CEM cells was reported upon treatment with extracts from some African plants. Amongst them were* Xylopia aethiopica* [40],* Anonidium mannii* [38], and* Polyscias fulva* [20]. The flavonoid** 22** and isoflavonoid** 23** induced very high ROS production, meanwhile high or moderate increases were measured with pterocarpan isoflavonoids** 28** and** 25** [53], 4′-hydroxy-2′,6′-dimethoxychalcone [56], and** 39** [19]. On the other hand, benzophenones** 19**–**21** were found not to increase ROS levels in leukemia CCRF-CEM cells [19].

### 7.5. Antiangiogenic Effects of African Plant Extract and Derived Molecules

Excessive angiogenesis represents an important pathogenic factor in many industrialized western countries [67]. Therefore, compounds with antiangiogenic properties are of importance in the treatment and prevention of malignancies as well as other chronic diseases [68, 69]. Herein, we recommend to classify the extend of inhibition of angiogenesis by plant extracts or derived molecules at not more than their twofold IC_50_ values as follows: (i) very strong: if the percentage of inhibition is more than 50%; (ii) strong: if the percentage of inhibition is between 20 and 50%; (iii) moderate: if the percentage of inhibition is between 10 and 20%; (iv) low: if the percentage of inhibition is between 5 and 10%; and (v) no induction: if the percentage of inhibition is below 5%.

The extracts from* Xylopia aethiopica*,* Dorstenia psilirus*,* Echinops giganteus,* and* Zingiber officinale* strongly inhibited angiogenesis in quail embryo [39]. A strong antiangiogenic activity on blood capillaries of the chorioallantoic membrane of quail eggs was also reported with compounds such as** 16**,** 36**, and** 17** [18, 57].

### 7.6. Other Modes of Action

Microarray analysis and signaling pathway profiling identified pathways and possible molecular targets involved in the cytotoxic effect of guieranone A (**36**) in leukemia CCRF-CEM cells. Several pathways and biological functions were affected by treatment** 36**, including the* cell cycle: G2/M DNA damage checkpoint regulation* and* ATM signaling* pathways [57]. The two most upregulated genes by** 36** were* HSPA6* (heat shock 70 kDa protein 6) and* HIST1H2BD* (histone cluster 1, H2bd). In cooperation with other chaperones,* HSPA6* stabilizes preexistent proteins against aggregation and mediates the folding of newly translated polypeptides in the cytosol as well as within organelles. They bind extended peptide segments with a net hydrophobic character exposed by polypeptides during translation and membrane translocation or following stress-induced damage [57]. Interestingly, 70 kDa heat shock protein protects cells from ischemia and its expression is increased in consequence to hypoglycemia [57], suggesting that** 36** might cause hypoxic stress. Histone H2B type 1D is a protein that is in humans encoded by the* HIST1H2BD* gene. Histones are basic nuclear proteins that are responsible for the nucleosome structure of the chromosomal fiber in eukaryotes. Levels of histone mRNA usually increase during S phase but decrease back to baseline level between the S phase and mitosis [57]. Certain histone mRNAs were upregulated after treatment with** 36** confirming the S phases cell cycle arrest by this compound [57]. Other upregulated genes were* FOSB* and* JUN*,* HIST2H2AC*,* HIST2H2AA4*, and CD52.

Significantly downregulated genes were* ACTB* and* ACTBL3*,* PGAM1*,* LOC728188*,* DHRS2*,* KPNA2*,* THOC4*,* RAB37* and* TRAPPC6A*,* HNRNPK*,* LYAR* and* YBX1*,* LYAR*,* YBX1*,* MYCN*, and* RUVBL1* [57].* ACTB* and* ACTBL3* belonged to the most downregulated genes. Beta-actin mRNA levels are known to be disturbed after ischemia [57], which is in line with the assumption that** 36** may mimic hypoxia. Another gene fitting to this hypothesis is* PGAM1*, which codes for phosphoglycerate mutase in glycolysis. Another gene coding for a protein similar to phosphoglycerate mutase processed protein was also downregulated by** 36**,* LOC728188* [57]. Downregulation of glycolysis key molecules accompanied by hypoxic stress may destroy the entire energy production aperture ultimately leading to cell death. The misregulation in glyco-related mechanisms by** 36** was also indicated by downregulation of* DHRS2*, whose encoded protein preferentially binds to glucose and related sugars [57].

*KPNA2* codes for importin alpha. This protein is a key player in the nuclear transport of macromolecules [57].* THOC4* encoding a more investigated mRNA transporter molecule was also significantly downregulated by** 36**. The THOC4 protein is part of the TREX complex, which specifically associates with spliced mRNA [57]. THOC4 is especially involved in nuclear export of Hsp70 transcripts [57]. Interestingly,* RAB37* and* TRAPPC6A* encode also two proteins which are also involved to transport mechanisms [57]. They were also misregulated in their transcriptional activity after** 36** treatment. In summary, transport mechanisms were deregulated as consequence of treatment of cancer cells with** 36** [57].

## 8. Structure-Activity Relationship of the Best Cytotoxic Compounds Identified in African Medicinal Plants

The general observation is that most of the terpenoids isolated from African medicinal plants such as compounds** 1**–**6** were more toxic on leukemia than on carcinoma cells [20, 37, 41, 50]. This observation is in accordance with the clinical situation, as it is well known that hematological tumors cells are frequently more sensitive than solid cancers [14]. Within the group of phenolics, this allegation varied, depending on the chemical structure. In fact, benzophenone moiety in compound** 18** and the polyisoprenylated compounds** 19**,** 20**, and** 21** were more active towards cell lines of different tumor types [19]. Furthermore,** 18** had the lowest activity, indicating that the polyprenylation and other substitutions in the cycle B influenced the antiproliferative capacity of benzophenones [19]. Amongst the three isomers (**19**,** 20,** and** 21**),** 19** and** 20** were more active than** 21** [19], suggesting that opening of the pyrone cycle in position C-31 may decrease the cytotoxic activity. The spatial configuration also influenced the cytotoxicity of benzophenones, as compounds** 19** and** 20** (two stereoisomers) revealed different degrees of activity on the majority of the studied cancer cell lines [19].

An analysis of the structure-activity relationship of flavonoids from* Polygonum limbatum* showed that the chalcones** 30**–**32** revealed considerable cytotoxicity in contrast to the flavanones** 33**–**35** [56]. The number of the hydroxyl (-OH) and methoxy (-OCH_3_) substituents influences the activity of chalcones towards leukemia as well as carcinoma cell lines. In fact, chalcone** 30** with two -OCH_3_ substituents (in positions C-2′ and C-6′) together with -OH group (in C-4′) demonstrated better activity than chalcones** 31** and** 32** with two -OH substituents and only one -OCH_3_ substituent [56]. The position of the -OH and -OCH_3_ did not significantly influence the activities of chalcones** 31** and** 32** [56].

## 9. Conclusion

In this review, we demonstrated that Africa flora contains several cytotoxic plants that could be used to fight MDR of cancer. The traditional use of the best plants (Table 1) indicated that they are not always used to treat cancers. Therefore, all plants independent of their ethnopharmacological relevance should be considered for cytotoxicity screenings in cancer cells. The most cytotoxic plant extracts from African flora were the spices* Aframomum arundinaceum, Xylopia aethiopica, Echinops giganteus, Imperata cylindrica, Piper capense, Dorstenia psilurus, *and* Zingiber officinale* as well as other medicinal plants such as* Uapaca togoensis, Gladiolus quartinianus, Beilschmiedia acuta, *and* Elaoephorbia drupifera*. The best cytotoxic phytochemicals were gancaonin Q (**12**), 6-prenylapigenin (**13**), 6,8-diprenyleriodictyol (**14**), 4-hydroxylonchocarpin (**15**), xanthone V_1_ (**16**), 2-acetylfuro-1,4-naphthoquinone (**17**), 2,2′,5,6′-tetrahydroxybenzophenone (**18**), isoxanthochymol (**20**), guttiferone E (**21**), 4′-hydroxy-2′,6′-dimethoxychalcone (**30**), guieranone A (**36**), isoneorautenol (**39**), cudraxanthone I (**42**), and arborinin (**44**). It can be concluded that African flora represents an enormous resource for the search of cytotoxic compounds. Intensified research efforts are warranted throughout the continent, for the development of novel anticancer drugs fighting MDR in the clinical setting.

## Figures and Tables

**Table 1:** African medicinal plants with demonstrated cytotoxicity on cancer cell lines.

Plant species (family)^*^/area of plant collection	Traditional use	Potential bioactive constituents	Reported cytotoxicity
*Acanthospermum hispidum* DC. (Asteraceae)/Nigeria	Cancer [70]	Hispidunolides A and B [71] and acanthospermal B [72]	Significant activity for roots methanol extract on COR-L23 (IC_50_: 8.87 *µ*g/mL) [70]

*Aframomum arundinaceum *(Rutaceae), spices/Cameroon	Laxative and, as antihelmintic, toothache fungal infections [73]	Aframodial, 8(17),12-labdadien-15,16-dial,galanolactone, 1-*p*-menthene-3,6-diol and 1,4-dimethoxybenzene, galanals A **(2) **and B **(3)**, naringenin **(7)**, and kaempferol-3,7,4′-trimethylether **(8)** [37]	Significant cytotoxicity of the crude extract on CCRF-CEM cells (IC_50_: 18.08 *µ*g/mL) and CEM/ADR5000 cells (IC_50_: 13.73 *µ*g/mL), MDA-MB231 cells (IC_50_: 29.98 *µ*g/mL), MDA-MB231/*BCRP* cells (IC_50_: 30.66 *µ*g/mL), HCT116(*p53* ^+/+^) cells (IC_50_: 23.06 *µ*g/mL), HCT116(*p53* ^−/−^) cells (IC_50_: 27.38 *µ*g/mL), U87MG cells (IC_50_: 36.70 *µ*g/mL), U87MG.Δ*EGFR * cells (IC_50_: 24.42 *µ*g/mL), and HepG2 cells (IC_50_: 23.15 *µ*g/mL) [37]. Moderate activity of compound **2** on CCRF-CEM cells (IC_50_: 17.32 *µ*M) and MDA-MB231/*BCRP* cells (IC_50_: 27.99 *µ*M), **3** on CCRF-CEM cells (IC_50_: 19.81 *µ*M), **7 **on CCRF-CEM cells (IC_50_: 12.20 *µ*M), CEM/ADR5000 cells (7.86 *µ*M), MDA-MB231 cells (IC_50_: 9.51 *µ*M), MDA- MDA-MB231/*BCRP* cells (IC_50_: 18.12 *µ*M), HCT116(*p53* ^+/+^) cells (IC_50_: 13.65 *µ*M), HCT116(*p53* ^−/−^) cells (IC_50_: 13.86 *µ*M), U87MG cells (IC_50_: 29.81 *µ*M), U87MG.Δ*EGFR* cells (IC_50_: 18.02 *µ*M), and HepG2 cells (IC_50_: 23.46 *µ*M) [37], and **8** on CCRF-CEM cells (IC_50_: 18.38 *µ*M), CEM/ADR5000 cells (IC_50_: 18.22 *µ*M), MDA-MB231/*BCRP* cells (IC_50_: 33.14 *µ*M), and HCT116(*p53* ^−/−^) cells (IC_50_: 36.74 *µ*M) [37]

*Aframomum melegueta *(Roscoe) K. Schum. (Rutaceae), edible plant/Cameroon	Constipation, fever, and carminative [74]	Volatile oil [75]	Significant activities with IC_50_ value above 10 *µ*g/mL on MiaPaca-2 and CCRF-CEM cells and significant activity of the crude extract on CEM/ADR5000 cells (IC_50_: 7.08 *µ*g/mL) [39]

*Aframomum polyanthum *(Rutaceae), spices/Cameroon	Bacterial infections and cancer [37]	Aframodial [73]	Significant activities of the crude extract on CCRF-CEM cells (IC_50_: 20.37 *µ*g/mL) and CEM/ADR5000 cells (IC_50_: 28.16 *µ*g/mL), MDA-MB231 cells (IC_50_: 33.79 *µ*g/mL), MDA-MB231/*BCRP* cells (IC_50_: 30.24 *µ*g/mL), and U87MG.Δ*EGFR *cells (IC_50_: 20.59 *µ*g/mL) [37]

*Albizia gummifera *(J. F. Gmel.) C. A. Sm. var. *gummifera* (Fabaceae)/Madagascar	Coughs, gonorrhea, fever, skin diseases, malaria, stomach pains, and wounds infection [76]	Gummiferaosides A, B, and C [53], budmunchiamine G, budmunchiamine K, 6′*ξ*-hydroxybudmunchiamine K, and 9-normethylbudmunchiamine K [76]	Significant activity for roots ethanol extract on A2780 cells (IC_50_: 7.2 *µ*g/mL)[46]

*Anonidium mannii* (Oliv.) Engl. et Diels. (Annonaceae), spices/Cameroon	Sore feet, spider bite, bronchitis, dysentery, sterility caused by poison, and gastroenteritis [77]; syphilis, infectious diseases [78]; diarrhea, snake bite, malaria [79], and cancer [38]	Alkaloids, phenols, tannins, and triterpenes [38]	Significant activity of the leaves crude extract on CCRF-CEM cells (IC_50_: 17.32 *µ*g/mL) and CEM/ADR5000 cells (IC_50_: 16.44 *µ*g/mL), MDA-MB231cells (IC_50_: 12.65 *µ*g/mL), MDA-MB231/*BCRP* cells (IC_50_: 32.02 *µ*g/mL), HCT116(*p53* ^+/+^) cells (IC_50_: 13.61 *µ*g/mL), U87MG cells (IC_50_: 22.25 *µ*g/mL), U87MG.Δ*EGFR * cells (IC_50_: 9.14 *µ*g/mL), and HepG2 cells (IC_50_: 22.09 *µ*g/mL) [38]

*Beilschmiedia acuta *Kosterm. (Lauraceae)/Cameroon	Cancer and gastrointestinal infections [20]	Flavonoids, phenols, saponins, and alkaloids [20]	Significant to moderate cytotoxicities of the leaves and bark extract on CCRF-CEM cells (IC_50_: 8.22 *µ*g/mL and 14.72 *µ*g/mL, resp.) and CEM/ADR5000 cells (IC_50_: 19.76 *µ*g/mL and 26.74 *µ*g/mL resp.), MDA-MB231 cells (IC_50_: 6.45 *µ*g/mL and 6.60 *µ*g/mL, resp.), MDA-MB231/*BCRP* cells (IC_50_: 21.01 *µ*g/mL and 22.75 *µ*g/mL resp.), HCT116(*p53* ^+/+^) cells (IC_50_: 21.12 *µ*g/mL and 11.62 *µ*g/mL, resp.), HCT116(*p53* ^−/−^) cells (IC_50_: 4.79 *µ*g/mL and 21.17 *µ*g/mL, resp.), U87MG cells (IC_50_: 7.46 *µ*g/mL and 7.27 *µ*g/mL, resp.), U87MG.Δ*EGFR *cells (IC_50_: 17.85 *µ*g/mL and 32.53 *µ*g/mL, resp.), and HepG2 cells (IC_50_: 23.09 *µ*g/mL for leaves extract) [20]

*Crinum zeylanicum *Linn. (Amaryllidaceae)/Cameroon	Rheumatism, earache, malaria, and poison [80]	Flexinine, 6-hydroxypowelline, zeylamine, hamayne, 3-acetylhamayne, crinamine, 6-hydroxycrinamine, 6-methoxycrinamine, crinine, ambelline, 6-hydroxybuphandrine, 6-ethoxybuphandrine, 6-ethoxybuphanidrine, lycorine, 11-*O*-acetoxyambelline, galantamine, sanguinine, and 3-*O*-acetylsanguinine [80]	Moderate activity of the whole plant extract on CEM/ADR5000 cells (IC_50_: 23.67 *µ*g/mL) and significant activity on CCRF-CEM cells (IC_50_: 17.22 *µ*g/mL), MDA-MB231 cells (IC_50_: 18.01 *µ*g/mL), MDA-MB231/*BCRP* cells (IC_50_: 11.18 *µ*g/mL), HCT116(*p53* ^+/+^) cells (IC_50_: 4.32 *µ*g/mL), and HCT116(*p53* ^−/−^) cells (IC_50_: 7.45 *µ*g/mL) [42]

*Dioscorea bulbifera *L.(Dioscoreaceae)/Cameroon	Sore throat and struma, leprosy and tumors, diabetes, and microbial infections [81, 82]	Kaempferol-3,5-dimethyl ether, caryatin, (+)-catechin, myricetin, quercetin-3-*O*-galactopyranoside, myricetin-3-*O*-galactopyranoside, myricetin-3-*O*-glucopyranoside, diosbulbin B [81], bafoudiosbulbins A, B, C, F, and G, and 2,7-dihydroxy-4-methoxyphenanthrene [82]	Moderate activity of the crude extract on MDA-MB231 cells (IC_50_: 33.17 *µ*g/mL), HCT116(*p53* ^−/−^) cells (IC_50_: 36.14 *µ*g/mL), and U87MG.Δ*EGFR * cells (IC_50_: 27.76 *µ*g/mL) and significant activity on CCRF-CEM cells (IC_50_: 19.77 *µ*g/mL) [42]

*Dorstenia psilurus *Welwitch (Moraceae)/Cameroon	Arthralgia, cardiovascular disorders, rheumatism, snakebites, headache, stomach disorders, diuretic, tonic, stimulant, analgesic [83–86], and spice	Psoralen and 2-sitosterol glucoside analgesic [84, 87]	Significant activity of the crude extract on MiaPaca-2 cells (IC_50_: 9.17 *µ*g/mL), CCRF-CEM cells (IC_50_: 7.18 *µ*g/mL), and CEM/ADR5000 cells (IC_50_: 7.79 *µ*g/mL) [39]

*Echinops giganteus* var. *lelyi *(C. D. Adams) A. Rich. (Compositae)/Cameroon	Heart and gastric troubles [88] and spice	Lupeol sitosterol, ß-D-glucopyranoside [44, 89–91], 2-(penta-1,3-diynyl)-5-(4-hydroxybut-1-ynyl)-thiophene **(49)**, candidone **(9)**, ursolic acid, and 4-hydroxy-2,6-di-(3′,4′-dimethoxyphenyl)-3,7dioxabicyclo-(3.3.0)octane **(10)** [40]	Significant activity of the crude extract on MiaPaca-2 cells (IC_50_: 9.84 *µ*g/mL), CCRF-CEM cells (IC_50_: 6.86 *µ*g/mL) and CEM/ADR5000 cells (IC_50_: 7.96 *µ*g/mL) [39], HL60 cells (IC_50_: 6.38 *µ*g/mL), HL60AR cells (IC_50_: 9.24 *µ*g/mL), MDA-MB231 cells (IC_50_: 8.61 *µ*g/mL), MDA-MB231/*BCRP* cells (IC_50_: 6.52 *µ*g/mL), HCT116(*p53* ^+/+^) cells (IC_50_: 3.58 *µ*g/mL), HCT116(*p53* ^−/−^) cells (IC_50_: 3.29 *µ*g/mL), U87MG cells (IC_50_: 113.55 *µ*g/mL), U87MG.Δ*EGFR * cells (IC_50_: 11.15 *µ*g/mL), and HepG2 cells (IC_50_: 14.32 *µ*g/mL) [40]; low activity ranged from 19 to 38 *µ*g/mL for compound **49** on all the 11 above cell lines and low and selective activities for **9** and **10** [40]

*Elaeodendron alluaudianum* H. Perrier (Celastraceae)/Madagascar	Not reported	Elaeodendrosides V and W and sarmentosigenin-3*β*-*O*-*β*-6-deoxyguloside [48]	Significant activity of the crude extract on A2780 cells (IC_50_: 3.3 *µ*g/mL) [48]

*Elaoephorbia drupifera *(Thonn.) Stapf. (Euphorbiaceae)/Cameroon	Hypertension and diabetes [92]	Euphol, tirucallol, euphorbol, ingenol elaeophorbate, epitaraxerol, taraxerone, friedelin, lup-20(29)-en-3-one or lupenone, lupeol, olean-12-ene-3-one, olean-12-ene-3-ol, and elaeophorbate [93, 94]	Moderate activity of the crude extract on CEM/ADR5000 cells (IC_50_: 26.14 *µ*g/mL), MDA-MB231/*BCRP* cells (IC_50_: 30.96 *µ*g/mL), HCT116(*p53* ^−/−^) cells (IC_50_: 25.36 *µ*g/mL), HCT116(*p53* ^+/+^) cells (IC_50_: 28.61 *µ*g/mL), U87MG cells (IC_50_: 23.58 *µ*g/mL), and HepG2 cells (IC_50_: 23.23 *µ*g/mL) and significant activity on CCRF-CEM cells (IC_50_: 11.86 *µ*g/mL), CEM/ADR5000 cells (IC_50_: 13.72 *µ*g/mL), MDA-MB231cells (IC_50_: 8.40 *µ*g/mL), and U87MG.Δ*EGFR *cells (IC_50_: 16.03 *µ*g/mL) [42]

*Entada abyssinica *Steud. ex A. Rich. (Mimosaceae)/Cameroon	Bronchitis, coughs, arthritic pains, miscarriage, fever, and abdominal pain [95]	Not reported	Moderate activity of the crude extract on MDA-MB231 (IC_50_: 29.14 *µ*g/mL) and significant activity on CCRF-CEM cells (IC_50_: 15.81 *µ*g/mL), HCT116(*p53* ^−/−^) cells (IC_50_: 9.55 *µ*g/mL), and HCT116(*p53* ^+/+^) cells (IC_50_: 14.38 *µ*g/mL) [42]

*Eremomastax speciosa *(Hochst.) Cufod. (Acanthaceae)/Cameroon	Dysentery, anemia, irregular menstruation, hemorrhoids, and urinary tract infection [96]	Not reported	Moderate activity of the crude extract on CCRF-CEM cells (IC_50_: 23.65 *µ*g/mL), CEM/ADR5000 cells (IC_50_: 38.71 *µ*g/mL), and MDA-MB231 cells (IC_50_: 35.13 *µ*g/mL) [42]

*Fagara leprieurii* (Guill. & Perr.) Engl. (Rutaceae)/Cameroon	Abdominal pain, asthma, appendicitis, toothache [97], and spice	3-Hydroxy-1-methoxy-10-methyl-9-acridone; 1-hydroxy-3-methoxy-10-methyl-9-acridone (4), 1-hydroxy-2,3-dimethoxy-10-methyl-9-acridone (5), and 1,3-dihydroxy-2-methoxy-10-methyl-9-acridone [61]	Significant activities with IC_50_ value above 10 *µ*g/mL on MiaPaca-2 cells and CCRF-CEM cells and significant activity of the crude extract on CEM/ADR5000 cells (IC_50_: 8.13 *µ*g/mL) [39]; compounds 3-hydroxy-1-methoxy-10-methyl-9-acridone, 1-hydroxy-3-methoxy-10-methyl-9-acridone, 1-hydroxy-2,3-dimethoxy-10-methyl-9-acridone, and 1,3-dihydroxy-2-methoxy-10-methyl-9-acridone were found to be moderately active (IC_50_ ranged from 27 to 77 *µ*M) on A549 and DLD-1 cells [61]

*Ferula hermonis *Chirch el. (Apiaceae)/Egypt	Skin infections, fever, dysentery, antihysteric, and aphrodisiac [98]	Jaeschkeanadiol *p*-hydroxybenzoate **(4)** [43]	Moderate activities of compound **4** on CCRF-CEM cells (IC_50_: 18.86 *µ*g/mL), CEM/ADR5000 cells (IC_50_: 19.92 *µ*g/mL), and MiaPaCa-2 cells (IC_50_: 10.22 *µ*g/mL) and significant activity on MCF-7 cells (IC_50_: 2.14 *µ*g/mL) [43]

*Gladiolus quartinianus *A. Rich (Iridaceae)/Cameroon	Gastrointestinal infections and cancer [38]	Alkaloids, anthocyanins, phenols, saponins, tannins, and triterpenes [38]	Moderate activity of the crude extract on CEM/ADR5000 cells (IC_50_: 26.14 *µ*g/mL), MDA-MB231/*BCRP* cells (IC_50_: 29.60 *µ*g/mL), HCT116(*p53* ^−/−^) cells (IC_50_: 22.15 *µ*g/mL), and U87MG.Δ*EGFR *cells (IC_50_: 34.01 *µ*g/mL) and significant activity on CCRF-CEM cells (IC_50_: 10.57 *µ*g/mL), MDA-MB231 cells (IC_50_: 16.11 *µ*g/mL), and HCT116(*p53* ^+/+^) cells (IC_50_: 19.83 *µ*g/mL) [38]

*Imperata cylindrica *Beauv. var. *koenigii* Durand et Schinz Gramineae (Poaceae), spice/Cameroon	Diuretic and anti-inflammatory agents [99] and spice	Jaceidin and quercetagetin-3, 5, 6, 3.′-tetramethyl ether; *β*-Sitosterol-3-0-*β*-D-glucopyranosy1-6′′- tetradecanoate [100]	Significant activity of the crude extract on MiaPaca-2 cells (IC_50_: 12.11 *µ*g/mL), CCRF-CEM cells (IC_50_: 8.4 *µ*g/mL) and CEM/ADR5000 cells (IC_50_: 7.18 *µ*g/mL) [39], HL60 cells (IC_50_: 11.30 *µ*g/mL), HL60AR cells (IC_50_: 26.64 *µ*g/mL), MDA-MB231 cells (IC_50_: 6.02 *µ*g/mL), MDA-MB231/*BCRP* cells (IC_50_: 13.08 *µ*g/mL), HCT116(*p53* ^+/+^) cells (IC_50_: 3.28 *µ*g/mL), HCT116(*p53* ^−/−^) cells (IC_50_: 4.32 *µ*g/mL), U87MG cells (IC_50_: 13.14 *µ*g/mL), U87MG.Δ*EGFR * cells (IC_50_: 14.79 *µ*g/mL), and HepG2 cells (IC_50_: 33.43 *µ*g/mL) [40]

*Olax subscorpioidea* var. *subscorpioidea* Oliv. (Olacaceae), spice/Cameroon	Constipation, yellow fever, jaundice, venereal diseases, Guinea worm [101], and spice	Santalbic acid [102, 103]	Significant activities with IC_50_ value above 10 *µ*g/mL on MiaPaca-2 and CCRF-CEM cells and significant activity of the crude extract on CEM/ADR5000 cells (IC_50_: 10.65 *µ*g/mL) [39]

*Piper capense* L.f. (Piperaceae), spice/Cameroon	sleep inducing remedy, anthelmintic [104, 105], and spice	Kaousine and Z-antiepilepsirine [106]	Significant activity of the crude extract on MiaPaca-2 cells (IC_50_: 8.92 *µ*g/mL), CCRF-CEM cells (IC_50_: 7.03 *µ*g/mL) and CEM/ADR5000 cells (IC_50_: 6.56 *µ*g/mL) [39], HL60 cells (IC_50_: 7.97 *µ*g/mL), HL60AR cells (IC_50_: 11.22 *µ*g/mL), MDA-MB231 cells (IC_50_: 4.17 *µ*g/mL), MDA-MB231/*BCRP* cells (IC_50_: 19.45 *µ*g/mL), HCT116(*p53* ^+/+^) cells (IC_50_: 4.67 *µ*g/mL), HCT116(*p53* ^−/−^) cells (IC_50_: 4.62 *µ*g/mL), U87MG (IC_50_: 13.48 *µ*g/mL), U87MG.Δ*EGFR* cells (IC_50_: 7.44 *µ*g/mL), and HepG2 cells (IC_50_: 16.07 *µ*g/mL) [40]

*Piper guineense *(Schum. and Thonn.) (Piperaceae), spice/Cameroon	Respiratory infections, female infertility, aphrodisiac [107], and spice	*N*-Isobutyl-ll-(3,4-methylenedioxyphenyl)-2*E*,4*E*,10*E*-undecatrienamide; N-pyrrolidyl-12-(3,4-methylene-dioxyphenyl)-2*E*,4*E*,9*E*,11*Z*-dodecatetraenamide; *N*-isobutyl-13-(3,4-methylenedioxyphenyl)-2*E*,4*E*,12*E*-tridecatrienamide; N-isobutyl-2*E*,4*E*-decadienamide; *N*-isobutyl-2*E*,4*E*-dodecadienamide [108]	Significant activities with IC_50_ value above 10 *µ*g/mL on MiaPaca-2 and CCRF-CEM cells and significant activity of the crude extract on CEM/ADR5000 cells (IC_50_: 8.20 *µ*g/mL) [39]

*Piliostigma thonningii* (Schum.) Milne-Redhead (Caesalpiniaceae)/Cameroon	Leprosy, smallpox, coughs, wounds, and ulcers [109]	Piliostigmin, quercetin, quercitrin, 6-C-methylquercetin 3-methyl ether, 6-C-methylquercetin 3,7,3′-trimethyl ether, 6,8-di-C-methylkaempferol 3-methyl ether, and 6,8-di-C-methylkaempferol 3,7-dimethyl ether [110]	Moderate activity of the crude extract on CCRF-CEM cells (IC_50_: 26.44 *µ*g/mL), MDA-MB231 cells (IC_50_: 34.19 *µ*g/mL), and U87MG cells (IC_50_: 34.22 *µ*g/mL) [42]

*Polyscias fulva* (Hiern) Harms. (Araliaceae)/Cameroon	Malaria, fever, mental illness [111]; venereal infections and obesity [112, 113], and cancer [20]	Polysciasoside A, kalopanax-saponin B, and alpha-hederin **(1)** [20, 114]	Significant to moderate cytotoxicities of the roots extract on CCRF-CEM cells (IC_50_: 7.79 *µ*g/mL) and CEM/ADR5000 cells (IC_50_: 22.63 *µ*g/mL), MDA-MB231 cells (IC_50_: 3.27 *µ*g/mL), MDA-MB231/*BCRP* cells (IC_50_: 16.67 *µ*g/mL), HCT116(*p53* ^+/+^) cells (IC_50_: 14.66 *µ*g/mL), HCT116(*p53* ^−/−^) cells (IC_50_: 5.98 *µ*g/mL), U87MG cells (IC_50_: 4.15 *µ*g/mL), U87MG.Δ*EGFR *cells (IC_50_: 16.35 *µ*g/mL), and HepG2 cells (IC_50_: 12.99 *µ*g/mL) [20]; moderate activity of its constituent **1** on CCRF-CEM cells (IC_50_: 6.29 *µ*M) and CEM/ADR5000 cells (IC_50_: 7.43 *µ*M), MDA-MB231 cells (IC_50_: 21.35 *µ*M), MDA-MB231/*BCRP* cells (IC_50_: 19.80 *µ*M), HCT116(*p53* ^+/+^) cells (IC_50_: 14.98 *µ*M), HCT116(*p53* ^−/−^) cells (IC_50_: 18.92 *µ*M), U87MG cells (IC_50_: 21.45 *µ*M), U87MG.Δ*EGFR* cells (IC_50_: 43.89 *µ*M), and HepG2 cells (IC_50_: 23.63 *µ*M) [20]

*Vepris soyauxii *Engl. (Rutaceae)/Cameroon	Antifibromyoma, stomachache, malaria [115], and cancer [38]	Alkaloids, anthocyanins, phenols, saponins, tannins, and triterpenes [38]	Moderate activity of the crude leaves extract on CEM/ADR5000 cells (IC_50_: 26.14 *µ*g/mL), MDA-MB231/*BCRP* cells (IC_50_: 29.60 *µ*g/mL), and HCT116(*p53* ^−/−^) cells (IC_50_: 22.15 *µ*g/mL) and significant to moderate cytotoxicities of the roots extract on CCRF-CEM cells (IC_50_: 10.57 *µ*g/mL), MDA-MB231 cells (IC_50_: 16.11 *µ*g/mL), and HCT116(*p53* ^+/+^) cells (IC_50_: 19.83 *µ*g/mL) [38]

*Uapaca togoensis*Pax.(Euphorbiaceae)/Cameroon	Skin disorders [116], pneumonia, cough, fever, rheumatism, vomiting, and epilepsy [117] and bacterial diseases [118]	*β*-Amyryl acetate, 11-oxo-*α*-amyryl acetate **(5)**, lupeol, pomolic acid, futokadsurin B **(11)**, arborinin **(44)**, and 3-*O-β-D*-glucopyranosyl sitosterol [41]	Moderate activity of the fruit extract on MDA-MB231 cells (IC_50_: 25.85 *µ*g/mL) and significant activity on CCRF-CEM cells (IC_50_: 4.23 *µ*g/mL) and CEM/ADR5000 cells (IC_50_: 4.44 *µ*g/mL), MDA-MB231/*BCRP *cells (IC_50_: 4.17 *µ*g/mL) and HCT116(*p53* ^+/+^) cells (IC_50_: 3.69 *µ*g/mL), HCT116(*p53* ^−/−^) cells (IC_50_: 3.09 *µ*g/mL), U87MG cells (IC_50_: 8.01 *µ*g/mL), U87MG.Δ*EGFR *cells (IC_50_: 8.68 *µ*g/mL), and HepG2 cells (IC_50_: 19.90 *µ*g/mL) [41]; compound **5** displayed selective activity on the above cell lines with significant effect on CCRF-CEM cells (IC_50_: 4.53 *µ*M) but low effect on CEM/ADR5000 cells (IC_50_: 78.93 *µ*M); compound **11** showed significant activity on CEM/ADR5000 cells (IC_50_: 8.16 *µ*M) and HepG2 (IC_50_: 10.85 *µ*M); **44** demonstrated significant activity on CEM/ADR5000 cells (IC_50_: 3.55 *µ*M), MDA-MB23 cells (IC_50_: 8.88 *µ*M), MDA-MB231/*BCRP* cells (IC_50_: 7.76 *µ*M) and HCT116(*p53* ^+/+^) cells (IC_50_: 6.01 *µ*M), HCT116(*p53* ^−/−^) cells (IC_50_: 8.67 *µ*M), U87MG.Δ*EGFR* cells (IC_50_: 6.89 *µ*M), and HepG2 cells (IC_50_: 7.10 *µ*M), moderate activity on U87MG cells (IC_50_: 20.41 *µ*M), and low activity on CCRF/CEM cells (IC_50_: 31.77 *µ*M) [41]

*Xylopia aethiopica* (Dunal) A. Rich. (Annonaceae), spice/Cameroon	Wounds and skin infections, fever, tapeworm, stomach ache, dysentery, stomach ulcer [119, 120], and spice	Volatile oil [121, 122]	Significant activity of the crude seeds extract on MiaPaca-2 cells (IC_50_: 6.86 *µ*g/mL), CCRF-CEM cells (IC_50_: 3.96 *µ*g/mL) and CEM/ADR5000 cells (IC_50_: 7.04 *µ*g/mL) [39], HL60 cells (IC_50_: 7.94 *µ*g/mL), HL60AR cells (IC_50_: 30.60 *µ*g/mL), MDA-MB231 cells (IC_50_: 5.19 *µ*g/mL), MDA-MB231/*BCRP* cells (IC_50_: 10.04 *µ*g/mL) and HCT116(*p53* ^+/+^) cells (IC_50_: 4.37 *µ*g/mL), HCT116(*p53* ^−/−^) cells (IC_50_: 4.60 *µ*g/mL), U87MG cells (IC_50_: 19.99 *µ*g/mL), U87MG.Δ*EGFR * cells (IC_50_: 10.68 *µ*g/mL), and HepG2 cells (IC_50_: 18.28 *µ*g/mL) [40]

*Zingiber officinale* Roscoe (Zingiberaceae), spice/Cameroon	Infectious diseases, respiratory tract infections, anticancer, indigestion, diarrhea, and nausea [123–125]	2-(4-Hydroxy-3-methoxyphenyl)ethanol and 2-(4-hydroxy-3-methoxyphenyl)ethanoic acid [124] and 6-shogaol [126]	Significant activity of the crude extract on MiaPaca-2 cells (IC_50_: 16.33 *µ*g/mL), CCRF-CEM cells (IC_50_: 8.82 *µ*g/mL), and CEM/ADR5000 cells (IC_50_: 6.83 *µ*g/mL); [39] reported cytotoxicity for 6-shogaol against human A549 cells, SK-OV-3 cells, SK-MEL-2 cells, and HCT15 ells [126]

Definition of cell lines [breast adenocarcinoma cells (MDA-MB231 and the resistant subline MDA-MB-231/*BCRP*, MCF7), colon cancer cells (DLD-1 and HCT15, HCT116*(p53* ^+/+^ *),* and the resistant subline HCT116*(p53* ^−/−^)), and glioblastoma multiforme (U87MG and the resistant subline U87MG.Δ*EGFR*)], hepatocarcinoma cells (HepG2), lung carcinoma cells (A549, COR-L23), leukemia cells (CCRF-CEM and the resistant subline CEM/ADR5000, HL60, and the resistant subline HL60AR), melanoma cells (SK-MEL-2), prostate cancer cells (MiaPaca-2), and ovarian cancer cells (A2780 and SK-OV-3); ^*^the criteria of classification of activities for spices as well as other edible plants are different from that of other plants as indicated in the text; galanals A** (2) **and B **(3)**, jaeschkeanadiol *p*-hydroxybenzoate **(4)**, 11-oxo-*α*-amyryl acetate **(5)**, naringenin** (7)**, kaempferol-3,7,4′-trimethylether **(8)**, futokadsurin B **(11)**, 2-(penta-1,3-diynyl)-5-(4-hydroxybut-1-ynyl)-thiophene **(49)**, candidone **(9)**, ursolic acid and 4-hydroxy-2,6-di-(3′,4′-dimethoxyphenyl)-3,7dioxabicyclo-(3.3.0)octane **(10)**, and arborinin **(44)**.

## Abbreviations

A2780 and SK-OV-3: Ovarian cancer cells
A549 and COR-L23: Lung carcinoma cells
ABC: Adenosine triphosphate-binding cassette
BCRP: Breast cancer resistance protein
CCRF-CEM and the resistant subline CEM/ADR5000, HL60, and the resistant subline HL60AR: Leukemia cells
D.R.: Degree of resistance
DLD-1, HCT15, HCT116 (*p*53^+/+^), and the resistant subline HCT116 (*p*53^−/−^): Colon cancer cells
DMAPP: Dimethylallyl diphosphate
EGFR: Epidermal growth factor receptor
GPP: Geranyl pyrophosphate
HepG2: Hepatocarcinoma cells
IARC: International Agency for Research on Cancer
IC_50_: Concentration inhibiting 50% of cell proliferation
IPP: Isopentenyl diphosphate
MCF7, MDA-MB231, and the resistant subline MDA-MB-231/*BCRP*: Breast adenocarcinoma cells
MDR: Multidrug resistant
MiaPaca-2: Prostate cancer cells
MMP: Mitochondrial membrane potential
p53: Tumor suppressor p53
Pgp: P-Glycoproteins
ROS: Reactive oxygen species
SK-MEL-2: Melanoma cells
TGF*α*: Transforming growth factor *α*
Topo: Topoisomerase
U87MG and the resistant subline U87MG.Δ*EGFR*: Glioblasma multiforme cells
WHO: World Health Organization.

## References

[1] http://www.cancer.org/acs/groups/content/@epidemiologysurveilance/documents/document/acspc-031574.pdf.

[2] Kuete V. (2013). *Medicinal Plant Research in Africa: Pharmacology and Chemistry*.

[3] Vorobiof D. A., Abratt R. (2007). The cancer burden in Africa. *South African Medical Journal*.

[4] Shen B., Li D., Dong P., Gao S. (2011). Expression of ABC transporters is an unfavorable prognostic factor in laryngeal squamous cell carcinoma. *Annals of Otology, Rhinology and Laryngology*.

[5] Biedler J. L., Spengler B. A. (1994). Reverse transformation of multidrug-resistant cells. *Cancer and Metastasis Reviews*.

[6] Efferth T., Sauerbrey A., Olbrich A. (2003). Molecular modes of action of artesunate in tumor cell lines. *Molecular Pharmacology*.

[7] Szakács G., Paterson J. K., Ludwig J. A., Booth-Genthe C., Gottesman M. M. (2006). Targeting multidrug resistance in cancer. *Nature Reviews Drug Discovery*.

[8] Efferth T. (2001). The human ATP-binding cassette transporter genes: from the bench to the bedside. *Current Molecular Medicine*.

[9] Gottesman M. M., Ling V. (2006). The molecular basis of multidrug resistance in cancer: the early years of P-glycoprotein research. *FEBS Letters*.

[10] Gillet J.-P., Efferth T., Remacle J. (2007). Chemotherapy-induced resistance by ATP-binding cassette transporter genes. *Biochimica et Biophysica Acta*.

[11] Eichhorn T., Efferth T. (2012). P-glycoprotein and its inhibition in tumors by phytochemicals derived from Chinese herbs. *Journal of Ethnopharmacology*.

[12] Ferlay J., Shin H., Bray F. (2010). GLOBO-CAN 2008. Cancer incidence and mortality worldwide. *IARC Cancer-Base*.

[13] World Health Organization (2008). *World Cancer Report 2008*.

[14] Kuete V., Efferth T. (2011). Pharmacogenomics of Cameroonian traditional herbal medicine for cancer therapy. *Journal of Ethnopharmacology*.

[15] Holmes K., Egan B., Swan N., O'Morain C. (2007). Genetic mechanisms and aberrant gene expression during the development of gastric intestinal metaplasia and adenocarcinoma. *Current Genomics*.

[16] Efferth T., Sauerbrey A., Halatsch M. E., Ross D. D., Gebhart E. (2003). Molecular modes of action of cephalotaxine and homoharringtonine from the coniferous tree *Cephalotaxus hainanensis* in human tumor cell lines. *Naunyn-Schmiedeberg's Archives of Pharmacology*.

[17] El-Deiry W. S. (1997). Role of oncogenes in resistance and killing by cancer therapeutic agents. *Current Opinion in Oncology*.

[18] Kuete V., Wabo H. K., Eyong K. O. (2011). Anticancer activities of six selected natural compounds of some Cameroonian medicinal plants. *PLoS ONE*.

[19] Kuete V., Tchakam P. D., Wiench B. (2013). Cytotoxicity and modes of action of four naturally occuring benzophenones: 2,2′,5,6′-Tetrahydroxybenzophenone, guttiferone E, isogarcinol and isoxanthochymol. *Phytomedicine*.

[20] Kuete V., Tankeo S. B., Saeed M. E. M., Wiench B., Tane P., Efferth T. (2014). Cytotoxicity and modes of action of five Cameroonian medicinal plants against multi-factorial drug resistance of tumor cells. *Journal of Ethnopharmacology*.

[21] Herbst R. S. (2004). Review of epidermal growth factor receptor biology. *International Journal of Radiation Oncology Biology Physics*.

[22] Yarden Y., Schlessinger J. (1987). Epidermal growth factor induces rapid, reversible aggregation of the purified epidermal growth factor receptor. *Biochemistry*.

[23] Downward J., Parker P., Waterfield M. D. (1984). Autophosphorylation sites on the epidermal growth factor receptor. *Nature*.

[24] Oda K., Matsuoka Y., Funahashi A., Kitano H. (2005). A comprehensive pathway map of epidermal growth factor receptor signaling. *Molecular Systems Biology*.

[25] Zhang H., Berezov A., Wang Q. (2007). ErbB receptors: from oncogenes to targeted cancer therapies. *The Journal of Clinical Investigation*.

[26] Walker F., Abramowitz L., Benabderrahmane D. (2009). Growth factor receptor expression in anal squamous lesions: modifications associated with oncogenic human papillomavirus and human immunodeficiency virus. *Human Pathology*.

[27] Matlashewski G., Lamb P., Pim D., Peacock J., Crawford L., Benchimol S. (1984). Isolation and characterization of a human p53 cDNA clone: expression of the human p53 gene. *The EMBO Journal*.

[28] Isobe M., Emanuel B. S., Givol D., Oren M., Croce C. M. (1986). Localization of gene for human p53 tumour antigen to band 17p13. *Nature*.

[29] Kern S. E., Kinzler K. W., Bruskin A. (1991). Identification of p53 as a sequence-specific DNA-binding protein. *Science*.

[30] Mitscher L. A. (2005). Bacterial topoisomerase inhibitors: quinolone and pyridone antibacterial agents. *Chemical Reviews*.

[31] Kuete V., Effeth T. (2014). *Biodiversity, Natural Products and Cancer Treatment*.

[32] United Nations Environment Programme (2009). *Biodiversity in Africa*.

[33] Stévigny C., Bailly C., Quetin-Leclercq J. (2005). Cytotoxic and antitumor potentialities of aporphinoid alkaloids. *Current Medicinal Chemistry - Anti-Cancer Agents*.

[34] Newman D. J., Cragg G. M. (2007). Natural products as sources of new drugs over the last 25 years. *Journal of Natural Products*.

[35] Voss C., Eyol E., Berger M. R. (2006). Identification of potent anticancer activity in *Ximenia americana* aqueous extracts used by African traditional medicine. *Toxicology and Applied Pharmacology*.

[36] Suffness M., Pezzuto J. (1990). *Assays Related to Cancer Drug Discovery*.

[37] Kuete V., Ango P. Y., Yeboah S. O. (2014). Cytotoxicity of four Aframomum species (*A. arundinaceum*, *A. alboviolaceum*, *A. kayserianum* and *A. polyanthum*) towards multi-factorial drug resistant cancer cell lines. *BMC Complementary and Alternative Medicine*.

[38] Kuete V., Fankam A. G., Wiench B., Efferth T. (2013). Cytotoxicity and modes of action of the methanol extracts of six cameroonian medicinal plants against multidrug-resistant tumor cells. *Evidence-Based Complementary and Alternative Medicine*.

[39] Kuete V., Krusche B., Youns M. (2011). Cytotoxicity of some Cameroonian spices and selected medicinal plant extracts. *Journal of Ethnopharmacology*.

[40] Kuete V., Sandjo L. P., Wiench B., Efferth T. (2013). Cytotoxicity and modes of action of four Cameroonian dietary spices ethno-medically used to treat cancers: *Echinops giganteus*, *Xylopia aethiopica*, *Imperata cylindrica* and *Piper capense*. *Journal of Ethnopharmacology*.

[41] Kuete V., Sandjo L., Seukep J. (2014). Cytotoxic compounds from the fruits of *Uapaca togoensis* towards multi-Factorial drug-resistant cancer cells. *Planta Medica*.

[42] Kuete V., Voukeng I. K., Tsobou R. (2013). Cytotoxicity of *Elaoephorbia drupifera* and other Cameroonian medicinal plants against drug sensitive and multidrug resistant cancer cells. *BMC Complementary and Alternative Medicine*.

[43] Kuete V., Wiench B., Hegazy M.-E. F. (2012). Antibacterial activity and cytotoxicity of selected Egyptian medicinal plants. *Planta Medica*.

[44] Kuete V., Wansi J. D., Mbaveng A. T. (2008). Antimicrobial activity of the methanolic extract and compounds from *Teclea afzelii* (Rutaceae). *South African Journal of Botany*.

[45] Mbaveng A. T., Hamm R., Kuete V., Kuete V. (2014). 19-Harmful and protective effects of terpenoids from African medicinal plants. *Toxicological Survey of African Medicinal Plants*.

[46] Cao S., Norris A., Miller J. S. (2007). Cytotoxic triterpenoid saponins of *Albizia gummifera* from the Madagascar rain forest. *Journal of Natural Products*.

[47] Williams R. B., Norris A., Miller J. S. (2007). Cytotoxic clerodane diterpenoids and their hydrolysis products from *Casearia nigrescens* from the rainforest of Madagascar. *Journal of Natural Products*.

[48] Hou Y., Cao S., Brodie P. (2009). Antiproliferative cardenolide glycosides of *Elaeodendron alluaudianum* from the Madagascar Rainforest. *Bioorganic & Medicinal Chemistry*.

[49] Rakotonandrasana O. L., Raharinjato F. H., Rajaonarivelo M. (2010). Cytotoxic 3,4- seco-atisane diterpenoids from *Croton barorum* and *Croton goudotii*. *Journal of Natural Products*.

[50] Sandjo L. P., Fru C. G., Kuete V. (2014). Elatumic acid: a new ursolic acid congener from *Omphalocarpum elatum* Miers (Sapotaceae). *Zeitschrift für Naturforschung C*.

[51] Mbaveng A. T., Zhao Q., Kuete V., Kuete V. (2014). Harmful and protective effects of phenolic compounds from African medicinal plants. *Toxicological Survey of African Medicinal Plants*.

[52] Kuete V., Ngameni B., Wiench B. (2011). Cytotoxicity and mode of action of four naturally occuring flavonoids from the genus *Dorstenia*: gancaonin Q, 4-hydroxylonchocarpin, 6-prenylapigenin, and 6,8-diprenyleriodictyol. *Planta Medica*.

[53] Kuete V., Sandjo L. P., Djeussi D. E. (2014). Cytotoxic flavonoids and isoflavonoids from *Erythrina sigmoidea* towards multi-factorial drug resistant cancer cells. *Investigational New Drugs*.

[54] Kuete V., Sandjo L. P., Kwamou G. M. N., Wiench B., Nkengfack A. E., Efferth T. (2014). Activity of three cytotoxic isoflavonoids from *Erythrina excelsa* and *Erythrina senegalensis* (neobavaisoflavone, sigmoidin H and isoneorautenol) toward multi-factorial drug resistant cancer cells. *Phytomedicine*.

[55] Kuete V., Sandjo L. P., Ouete J. L. N., Fouotsa H., Wiench B., Efferth T. (2014). Cytotoxicity and modes of action of three naturally occurring xanthones (8-hydroxycudraxanthone G, morusignin i and cudraxanthone I) against sensitive and multidrug-resistant cancer cell lines. *Phytomedicine*.

[56] Kuete V., Nkuete A. H. L., Mbaveng A. T. (2014). Cytotoxicity and modes of action of 4′-hydroxy-2′,6′-dimethoxychalcone and other flavonoids toward drug-sensitive and multidrug-resistant cancer cell lines. *Phytomedicine*.

[57] Kuete V., Eichhorn T., Wiench B., Krusche B., Efferth T. (2012). Cytotoxicity, anti-angiogenic, apoptotic effects and transcript profiling of a naturally occurring naphthyl butenone, guieranone A. *Cell Division*.

[58] Kwamou G. M., Sandjo L. P., Kuete V. (2014). Unprecedented new nonadecyl para-hydroperoxycinnamate isolated from *Erythrina excelsa* and its cytotoxic activity. *Natural Product Research: Formerly Natural Product*.

[59] Kuete V., Kuete V. (2014). 22—physical, hematological, and histopathological signs of toxicity induced by African medicinal plants. *Toxicological Survey of African Medicinal Plants*.

[60] Kuete V., Kuete V. (2014). 21-Health effects of alkaloids from African medicinal plants. *Toxicological Survey of African Medicinal Plants*.

[61] Ngoumfo R. M., Jouda J.-B., Mouafo F. T. (2010). *In vitro* cytotoxic activity of isolated acridones alkaloids from *Zanthoxylum leprieurii* Guill. et Perr. *Bioorganic and Medicinal Chemistry*.

[62] Sandjo L. P., Kuete V., Tchangna R. S., Efferth T., Ngadjui B. T. (2014). Cytotoxic benzophenanthridine and furoquinoline alkaloids from *Zanthoxylum buesgenii* (Rutaceae). *Chemistry Central Journal*.

[63] Alnemri E. S., Livingston D. J., Nicholson D. W. (1996). Human ICE/CED-3 protease nomenclature. *Cell*.

[64] Dejean L. M., Martinez-Caballero S., Kinnally K. W. (2006). Is MAC the knife that cuts cytochrome c from mitochondria during apoptosis?. *Cell Death and Differentiation*.

[65] Raj L., Ide T., Gurkar A. U. (2011). Selective killing of cancer cells by a small molecule targeting the stress response to ROS. *Nature*.

[66] Yap T. A., Sandhu S. K., Carden C. P., De Bono J. S. (2011). Poly (ADP-ribose) polymerase (PARP) inhibitors: exploiting a synthetic lethal strategy in the clinic. *CA Cancer Journal for Clinicians*.

[67] Krenn L., Paper D. H. (2009). Inhibition of angiogenesis and inflammation by an extract of red clover (*Trifolium pratense* L.). *Phytomedicine*.

[68] Paper D. H. (1998). Natural products as angiogenesis inhibitors. *Planta Medica*.

[69] Carmeliet P. (2003). Angiogenesis in health and disease. *Nature Medicine*.

[70] Ashidi J. S., Houghton P. J., Hylands P. J., Efferth T. (2010). Ethnobotanical survey and cytotoxicity testing of plants of South-western Nigeria used to treat cancer, with isolation of cytotoxic constituents from *Cajanus cajan* Millsp. leaves. *Journal of Ethnopharmacology*.

[71] Cartagena E., Bardon A., Catalan C. A. N., De Hernadez Z. N. J., Hernandez L. R., Joseph-Nathan P. (2000). Germacranolides and a new type of guaianolide from *Acanthospermum hispidum*. *Journal of Natural Products*.

[72] Arena M. E., Cartagena E., Gobbato N., Baigori M., Valdez J. C., Bardon A. (2011). *In vivo* and *in vitro* antibacterial activity of acanthospermal B, a sesquiterpene lactone isolated from *Acanthospermum hispidum*. *Phytotherapy Research*.

[73] Tane P., Tatsimo S., Ayimele G., Connolly J. Bioactive metabolites from *Aframomum* species.

[74] Dalziel J. (1936). *The Useful Plants of West Tropical Africa*.

[75] Oloke J. K., Kolawole D. O., Erhun W. O. (1998). The antibacterial and antifungal activities of certain components of *Aframomum melegueta* fruits. *Fitoterapia*.

[76] Rukunga G. M., Waterman P. G. (1996). New macrocyclic spermine (budmunchiamine) alkaloids from *Albizia gummifera*: with some observations on the structure—activity relationships of the budmunchiamines. *Journal of Natural Products*.

[77] Thomas J., Bahuchets S., Epelboin A., Fürniss S. (2003). *Encyclopédie des Pygmées Aka:techniques, langage et société des chasseurs-cueilleurs de la forêt centrafricaine(Sud-Centrafrique et Nord-Congo)*.

[78] Noumi E., Eloumou M. (2011). Syphilis ailment: prevalence and herbal remedies in Ebolowa subdivision (South region, Cameroon). *International Journal of Biomedical and Pharmaceutical Sciences*.

[79] Betti J. (2004). An ethnobotanical study of medicinal plants among the Baka Pygmies in the Dja Biosphere Reserve, Cameroon. *African Study Monographs*.

[80] Berkov S., Romani S., Herrera M. (2011). Antiproliferative alkaloids from *Crinum zeylanicum*. *Phytotherapy Research*.

[81] Gao H., Kuroyanagi M., Wu L., Kawahara N., Yasuno T., Nakamura Y. (2002). Antitumor-promoting constituents from *Dioscorea bulbifera* L. in JB6 mouse epidermal cells. *Biological and Pharmaceutical Bulletin*.

[82] Kuete V., BetrandTeponno R., Mbaveng A. T. (2012). Antibacterial activities of the extracts, fractions and compounds from *Dioscorea bulbifera*. *BMC Complementary and Alternative Medicine*.

[83] Ruppelt B. M., Pereira E. F., Gonçalves L. C., Pereira N. A. (1991). Pharmacological screening of plants recommended by folk medicine as anti-snake venom—I. Analgesic and anti-inflammatory activities. *Memorias do Instituto Oswaldo Cruz*.

[84] Adjanohoun J., Aboubakar N., Dramane K. (1996). *Traditional Medicine and Pharmacopoeia: Contribution to Ethnobotanical and Floristic Studies in Cameroon*.

[85] Ngadjui B. T., Dongo E., Happi E. N., Bezabih M.-T., Abegaz B. M. (1998). Prenylated flavones and phenylpropanoid derivatives from roots of *Dorstenia psilurus*. *Phytochemistry*.

[86] Dimo T., Rakotonirina A., Tan P. V. (2001). Antihypertensive effects of *Dorstenia psilurus* extract in fructose-fed hyperinsulinemic, hypertensive rats. *Phytomedicine*.

[87] Kuete V., Metuno R., Ngameni B. (2007). Antimicrobial activity of the methanolic extracts and compounds from *Treculia obovoidea* (Moraceae). *Journal of Ethnopharmacology*.

[88] Tene M., Tane P., Sondengam B. L., Connolly J. D. (2004). Lignans from the roots of *Echinops giganteus*. *Phytochemistry*.

[89] Kojima H., Sato N., Hatano A., Ogura H. (1990). Sterol glucosides from *Prunella vulgaris*. *Phytochemistry*.

[90] Tane P., Bergquist K.-E., Téné M., Ngadjui B. T., Ayafor J. F., Sterner O. (1995). Cyclodione, an unsymmetrical dimeric diterpene from *Cylicodiscus gabunensis*. *Tetrahedron*.

[91] Kuete V., Eyong K. O., Folefoc G. N. (2007). Antimicrobial activity of the methanolic extract and of the chemical constituents isolated from *Newbouldia laevis*. *Pharmazie*.

[92] Eno A. E., Azah N. (2004). Effect of ethanolic extract from *Elaeophorbia drupifera* leaves on the gastrointestinal smooth muscle of the rabbit. *Nigerian Journal of Physiological Sciences*.

[93] Kinghorn A. D., Evans F. J. (1974). Occurrence of ingenol in *Elaeophorbia* species. *Planta Medica*.

[94] Ahiahonu P. W. K., Goodenowe D. B. (2007). Triterpenoids from leaves of *Elaeophorbia drupifera*. *Fitoterapia*.

[95] Olajide O. A., Alada A. R. A. (2001). Studies on the anti-inflammatory properties of *Entada abyssinica*. *Fitoterapia*.

[96] Oben J. E., Assi S. E., Agbor G. A., Musoro D. F. (2006). Effect of *Eremomastax speciosa* on experimental diarrhoea. *African Journal of Traditional, Complementary and Alternative Medicines*.

[97] Diniz M., Martins E., Gomes E. (2007). Contribution to the knowledge of medicinal plants from Guinea-Bissau. *Portugaliae Acta Biologica*.

[98] Galal A. M., Abourashed E. A., Ross S. A., ElSohly M. A., Al-Said M. S., El-Feraly F. S. (2001). Daucane sesquiterpenes from *Ferula hermonis*. *Journal of Natural Products*.

[99] Nishimoto K., Ito M., Natori S., Ohmoto T. (1968). The structures of arundoin, cylindrin and fernenol: triterpenoids of fernane and arborane groups of *Imperata cylindrica* var. *Koenigii*. *Tetrahedron*.

[100] Mohamed G. A., Abdel-Lateff A., Fouad M. A., Ibrahim S. R. M., Elkhayat E. S., Okino T. (2009). Chemical composition and hepato-protective activity of *Imperata cylindrica* Beauv. *Pharmacognosy Magazine*.

[101] Okoli R. I., Aigbe O., Ohaju-Obodo J. O., Mensah J. K. (2007). Medicinal herbs used for managing some common ailments among Esan people of Edo State, Nigeria. *Pakistan Journal of Nutrition*.

[102] Jones G. P., Sundar Rao K., Tucker D. J., Richardson B., Barnes A., Rivett D. E. (1995). Antimicrobial activity of santalbic acid from the oil of *Santalum acuminatum* (Quandong). *International Journal of Pharmacognosy*.

[103] Cantrell C. L., Berhow M. A., Phillips B. S., Duval S. M., Weisleder D., Vaughn S. F. (2003). Bioactive crude plant seed extracts from the NCAUR oilseed repository. *Phytomedicine*.

[104] Kokowaro J. (1976). *Medicinal Plants of East Africa*.

[105] van Wyk B., Gericke N. (2000). *Peoples Plants: A Guide to Useful Plants in Southern Africa*.

[106] Kaou A. M., Mahiou-Leddet V., Canlet C. (2010). New amide alkaloid from the aerial part of *Piper capense* L.f. (Piperaceae). *Fitoterapia*.

[107] Noumi E., Zollo P. H. A., Lontsi D. (1998). Aphrodisiac plants used in Cameroon. *Fitoterapia*.

[108] Gbewonyo W. S. K., Candy D. J. (1992). Chromatographic isolation of insecticidal amides from *Piper guineense* root. *Journal of Chromatography A*.

[109] Akinpelu D. A., Obuotor E. M. (2000). Antibacterial activity of *Piliostigma thonningii* stem bark. *Fitoterapia*.

[110] Ibewuike J. C., Ogundaini A. O., Ogungbamila F. O. (1996). Piliostigmin, a 2-phenoxychromone, and C-methylflavonol from *Piliostigma thonningii*. *Phytochemistry*.

[111] Tshibangu J. N., Chifundera K., Kaminsky R., Wright A. D., König G. M. (2002). Screening of African medicinal plants for antimicrobial and enzyme inhibitory activity. *Journal of Ethnopharmacology*.

[112] Jeruto P., Lukhoba C., Ouma G., Otieno D., Mutai C. (2008). Herbal treatments in Aldai and Kaptumo divisions in Nandi district, Rift valley province, Kenya. *African Journal of Traditional, Complementary and Alternative Medicines*.

[113] Focho D. A., Ndam W. T., Fonge B. A. (2009). Medicinal plants of Aguambu—Bamumbu in the Lebialem highlands, southwest province of Cameroon. *African Journal of Pharmacy and Pharmacology*.

[114] Bedir E., Toyang N. J., Khan I. A., Walker L. A., Clark A. M. (2001). A new dammarane-type triterpene glycoside from *Polyscias fulva*. *Journal of Natural Products*.

[115] Momeni J., Ntchatchoua W. P. D. D., Akam M. T., Ngassoum M. B. (2010). Antioxidant activities of some cameroonian plants extracts used in the treatment of intestinal and infectious diseases. *Indian Journal of Pharmaceutical Sciences*.

[116] Mengome L.-E., Akue J. P., Souza A., Tchoua G. R. F., Emvo E. N. (2010). *In vitro* activities of plant extracts on human Loa loa isolates and cytotoxicity for eukaryotic cells. *Parasitology Research*.

[117] Koné W. M., Kamanzi Atindehou K., Kacou-N'Douba A., Dosso M. (2006). Evaluation of 17 medicinal plants from Northern Côte d'Ivoire for their *in vitro* activity against *Streptococcus pneumoniae*. *African Journal of Traditional, Complementary and Alternative Medicines*.

[118] Koné W. M., Atindehou K. K., Terreaux C., Hostettmann K., Traoré D., Dosso M. (2004). Traditional medicine in North Côte-d'Ivoire: screening of 50 medicinal plants for antibacterial activity. *Journal of Ethnopharmacology*.

[119] Irvine R. (1961). *Woody Plant of Ghana*.

[120] Thomas S. (1965). Chemical basis of drug action. *Drug Plants of Africa*.

[121] Tatsadjieu L. N., Ngang J. J. E., Ngassoum M. B., Etoa F.-X. (2003). Antibacterial and antifungal activity of *Xylopia aethiopica*, *Monodora myristica*, *Zanthoxylum xanthoxyloïdes* and *Zanthoxylum leprieurii* from Cameroon. *Fitoterapia*.

[122] Konan N., Kouame B. A., Mamyrbekova-Bekro J. A., Nemlin J., Yves-Alain B. (2009). Chemical composition and antioxidant activities of essential oils of *Xylopia aethiopica* (Dunal) a. rich. *European Journal of Scientific Research*.

[123] Akoachere J.-F. T. K., Ndip R. N., Chenwi E. B., Ndip L. M., Njock T. E., Anong D. N. (2002). Antibacterial effect of *Zingiber officinale* and *Garcinia kola* on respiratory tract pathogens. *East African Medical Journal*.

[124] Kato A., Higuchi Y., Goto H. (2006). Inhibitory effects of *Zingiber officinale* roscoe derived components on aldose reductase activity *in vitro* and *in vivo*. *Journal of Agricultural and Food Chemistry*.

[125] Sakpakdeejaroen I., Itharat A. (2009). Cytotoxic compounds against breast adenocarcinoma cells (MCF-7) from Pikutbenjakul. *Journal of Health Research*.

[126] Kim J. S., Lee S. I., Park H. W. (2008). Cytotoxic components from the dried rhizomes of *Zingiber officinale* Roscoe. *Archives of Pharmacal Research*.

